# Mustn1 is a smooth muscle cell-secreted microprotein that modulates skeletal muscle extracellular matrix composition

**DOI:** 10.1016/j.molmet.2024.101912

**Published:** 2024-03-06

**Authors:** Serge Ducommun, Paulo R. Jannig, Igor Cervenka, Marta Murgia, Melanie J. Mittenbühler, Ekaterina Chernogubova, José M. Dias, Baptiste Jude, Jorge C. Correia, Jonathan G. Van Vranken, Gabriel Ocana-Santero, Margareta Porsmyr-Palmertz, Sarah McCann Haworth, Vicente Martínez-Redondo, Zhengye Liu, Mattias Carlström, Matthias Mann, Johanna T. Lanner, Ana I. Teixeira, Lars Maegdefessel, Bruce M. Spiegelman, Jorge L. Ruas

**Affiliations:** 1 Molecular and Cellular Exercise Physiology, Department of Physiology and Pharmacology, Biomedicum, Karolinska Institutet, 171 77 Stockholm, Sweden; 2 Department of Biomedical Sciences, University of Padova, Via Ugo Bassi, 58/B, 35131 Padua, Italy; 3 Max-Planck-Institute of Biochemistry, Am Klopferspitz 18, 82152 Martinsried, Germany; 4 Department of Cancer Biology, Dana-Farber Cancer Institute, Boston, MA 02115, USA; 5 Department of Cell Biology, Harvard Medical School, Boston, MA 02115, USA; 6 Department of Medicine, Cardiovascular Unit, Karolinska Institutet, 171 77 Stockholm, Sweden; 7 Department of Cell and Molecular Biology, Biomedicum, Karolinska Institutet, 171 77 Stockholm, Sweden; 8 Nanomedicine and Spatial Biology, Department of Physiology and Pharmacology, Biomedicum, Karolinska Institutet, 171 77 Stockholm, Sweden; 9 Molecular Muscle Physiology and Pathophysiology, Department of Physiology and Pharmacology, Biomedicum, Karolinska Institutet, 171 77 Stockholm, Sweden; 10 Department of Physiology and Pharmacology, Biomedicum, Karolinska Institutet, 171 77 Stockholm, Sweden; 11 Institute of Molecular Vascular Medicine, Klinikum rechts der Isar, Technical University of Munich, 81675 Munich, Germany; 12 German Center for Cardiovascular Research DZHK, Partner Site Munich Heart Alliance, 10785 Berlin, Germany; 13 Department of Pharmacology and Stanley and Judith Frankel Institute for Heart & Brain Health, University of Michigan Medical School, Ann Arbor, MI 48109, USA

**Keywords:** Mustn1, Microprotein, Smooth muscle, Muscle regeneration, Extracellular matrix, Leiokine

## Abstract

**Objective:**

Skeletal muscle plasticity and remodeling are critical for adapting tissue function to use, disuse, and regeneration. The aim of this study was to identify genes and molecular pathways that regulate the transition from atrophy to compensatory hypertrophy or recovery from injury. Here, we have used a mouse model of hindlimb unloading and reloading, which causes skeletal muscle atrophy, and compensatory regeneration and hypertrophy, respectively.

**Methods:**

We analyzed mouse skeletal muscle at the transition from hindlimb unloading to reloading for changes in transcriptome and extracellular fluid proteome. We then used qRT-PCR, immunohistochemistry, and bulk and single-cell RNA sequencing data to determine Mustn1 gene and protein expression, including changes in gene expression in mouse and human skeletal muscle with different challenges such as exercise and muscle injury. We generated Mustn1-deficient genetic mouse models and characterized them *in vivo* and *ex vivo* with regard to muscle function and whole-body metabolism. We isolated smooth muscle cells and functionally characterized them, and performed transcriptomics and proteomics analysis of skeletal muscle and aorta of Mustn1-deficient mice.

**Results:**

We show that *Mustn1* (Musculoskeletal embryonic nuclear protein 1, also known as Mustang) is highly expressed in skeletal muscle during the early stages of hindlimb reloading. *Mustn1* expression is transiently elevated in mouse and human skeletal muscle in response to intense exercise, resistance exercise, or injury. We find that *Mustn1* expression is highest in smooth muscle-rich tissues, followed by skeletal muscle fibers. Muscle from heterozygous Mustn1-deficient mice exhibit differences in gene expression related to extracellular matrix and cell adhesion, compared to wild-type littermates. Mustn1-deficient mice have normal muscle and aorta function and whole-body glucose metabolism. We show that Mustn1 is secreted from smooth muscle cells, and that it is present in arterioles of the muscle microvasculature and in muscle extracellular fluid, particularly during the hindlimb reloading phase. Proteomics analysis of muscle from Mustn1-deficient mice confirms differences in extracellular matrix composition, and female mice display higher collagen content after chemically induced muscle injury compared to wild-type littermates.

**Conclusions:**

We show that, in addition to its previously reported intracellular localization, Mustn1 is a microprotein secreted from smooth muscle cells into the muscle extracellular space. We explore its role in muscle ECM deposition and remodeling in homeostasis and upon muscle injury. The role of Mustn1 in fibrosis and immune infiltration upon muscle injury and dystrophies remains to be investigated, as does its potential for therapeutic interventions.

## Highlights

• *Mustn1* expression increases during muscle remodeling.
• Mustn1 is expressed in arterioles of the muscle microvasculature.
• Mustn1 is secreted by smooth muscle cells.
• Loss of Mustn1 changes muscle extracellular matrix composition.

## 1 Introduction

Skeletal muscle is a highly adaptive tissue that changes with many physiological stimuli such as exercise, disuse, weightlessness, age, and is indispensable for locomotion, posture, and breathing. It is composed of many different cell types, which contribute to whole-body energy homeostasis [1] and serve endocrine functions [2]. Its parenchyma, the skeletal muscle fiber, mediates the contractile function of this organ, while other cell types such as satellite cells (muscle stem cells), fibro-adipogenic progenitors (FAPs), resident immune cells or vascular cells contribute to its function in homeostasis and regeneration. For tissue repair to occur after muscle injury, the concerted action of all these cell types is required to proceed through the process of inflammation, extracellular matrix (ECM) remodeling, and restoration of function [3]. Fibroblasts and FAPs are the main contributors to ECM deposition, which is necessary to preserve the integrity of the muscle while damaged fibers are cleared. Together with cells of the muscle microvasculature FAPs signal to satellite cells during activation and return to quiescence [4]. Vascular cells include endothelial cells, smooth muscle cells (SMCs) and pericytes, although the latter are relatively rare within striated muscle [5]. Endothelial cells, which line all the blood vessels, including capillaries, are necessary for the revascularization (angiogenesis) of damaged tissue [6]. They provide signals to skeletal muscle fibers and precursor cells [7], and immune cells [8]. How SMCs are affected by muscle injury and disease, and how they signal to their neighboring cells is less well known. SMCs constitute the medial layer of arteries, arterioles, and veins (to a lesser extent also venules), and their function ranges from contraction to regulate vessel diameter and blood flow [9,10] to synthesis of ECM components, giving the blood vessels structural support [11]. Moreover, their central role in diseases such as atherosclerosis is now also evident, where they have been shown to undergo phenotypic modulation from a contractile to a synthetic (secretory) phenotype [12]. Their contribution to muscle ECM deposition in homeostasis as well as their role in ECM remodeling upon muscle injury however is poorly understood.

To uncover novel genes and molecular pathways important for skeletal muscle remodeling and regeneration, we used a mouse hindlimb unloading and reloading protocol. We then performed analysis of global gene expression by RNA-sequencing (RNA-seq), looking at the transition from muscle unloading to reloading. This study focuses on the microprotein Mustn1 (Musculoskeletal embryonic nuclear protein 1, also known as Mustang), which consists of 82 amino acids. Microproteins (or micropeptides), defined as proteins encoded by a small (<100 amino acids) open reading frame (ORF), have recently garnered considerable attention as it was shown that they can have fundamental biological functions despite lacking apparent functional domains [13]. Although the *Mustn1* gene is known to be expressed in skeletal muscle during embryonic development and regeneration [14], little is known about its physiological function. Previous studies have suggested a role in myoblast differentiation [15,16], muscle function [17], and whole-body glucose homeostasis [18]. Here, we show that Mustn1 is expressed and secreted from SMCs into the muscle extracellular space. Thus, we suggest the term “leiokine” (derived from “leios”, the Greek word for smooth) to describe proteins such as Mustn1 that are secreted primarily from SMCs. Furthermore, we explore the role of Mustn1 in ECM deposition and remodeling in homeostasis and upon muscle injury.

## 2 Materials and methods

### 2.1 Animals

All animal experiments were approved by the regional animal ethics committee of Northern Stockholm, Sweden (Permits 4039-2018 and 4359-2020 to Jorge Ruas). All mice were housed at ca. 22 °C on a 12-hour light/dark cycle. Unless otherwise stated, mice were housed in groups of 3–5 per cage (GM500 cages, Tecniplast), with free access to water and food (Special Diet Services CRM-P). Wildtype mice (C57BL/6N and C57BL/6J) were obtained from Janvier Labs. Mustn1 KO-first mice (C57BL/6N-A^tm1Brd^ Mustn1^tm1a(EUCOMM)Wtsi^/BayMmucd) were obtained from MMRRC (ref. 041556-UCD) and negatively selected against the tyrosinase albino (Tyr^c^) allele. Intercrossing of Mustn1^+/tm1a^ mice produced viable offspring at expected Mendelian ratio (analyzed offspring, n = 95; Mustn1^+/+^, n = 22 (23.16%), Mustn1^+/tm1a^, n = 48 (50.53%) and Mustn1^tm1a/tm1a^, n = 25 (26.32%)). Myog-Cre mice were obtained from Dr. Eric Olson (UT Southwestern) [19]. Actb-Cre and Flp deleter mice (C57BL/6N-Tg(ACTB-FLP)Dym) were a kind gift from Dr. Nils-Göran Larsson (Karolinska Institutet, Sweden). All mice were maintained on a C57BL/6N background and heterozygous male and female mice were bred for experimental cohorts, unless otherwise stated.

### 2.2 Generation of whole-body and skeletal muscle-specific Mustn1 KO mice

Mice carrying the KO-first allele were crossed with a Beta-Actin promoter-driven Flp recombinase line (Actb-Flp) to remove the LacZ and neomycin cassettes, thus generating the Mustn1 conditional-ready (“floxed”) allele (tm1c) [20]. For the muscle-specific Mustn1 KO experiments, homozygous “floxed” females (tm1c/tm1c) were bred with heterozygous “floxed” males carrying the MEF2C/Myogenin promoter-Cre recombinase gene (tm1c/wt, Myog-Cre+). To generate the whole-body Mustn1 KO mice, mice carrying the tm1c allele were crossed with Actb-Cre mice to delete exon 2 and a part of exon 3 of the *Mustn1* gene. Offspring were selected for germline transmission of the KO allele (tm1d) and further bred to remove the Actb-Cre recombinase gene. Intercrossing of Mustn1^+/tm1d^ mice produced viable offspring at expected Mendelian ratio (analyzed offspring, n = 290; Mustn1^+/+^, n = 73 (25.17%), Mustn1^+/tm1d^, n = 140 (48.28%) and Mustn1^tm1d/tm1d^, n = 77 (26.55%)).

### 2.3 Hindlimb unloading/reloading

For the hindlimb unloading procedure, cages (GR900, Tecniplast) were equipped with two parallel aluminum rods running along the top of the cage. Female mice were restrained, and a nylon line was attached to the base of their tail using medical tape. The nylon line was connected to a swivel and ring and attached to the aluminum rod. The length of the nylon line was adjusted to slightly elevate the hindquarters and prevent the hindlimbs from bearing weight, while allowing ambulation using the forelimbs. Two mice were housed per cage for the duration of the unloading procedure (up to 10 days), with *ad libitum* access to water and food. For the reloading phase, mice were returned to their home cage after removal of the nylon line and tape. Control mice were housed in conventional cages for the duration of the procedure.

### 2.4 Chemically induced muscle injury

Mice were anaesthetized using isofluorane and one leg was injected with BaCl_2_ (Sigma–Aldrich ref. B0750, 1.2 % in phosphate-buffered saline) or cardiotoxin (CTX, Sigma–Aldrich ref. 217,503, 10 μM in phosphate-buffered saline) using a 30 G needle, while the other leg (contra-lateral control) was not injected. For the BaCl_2_ experiments, gastrocnemius muscle was injected with 50 μl, whereas for the CTX experiments, 30 μl were injected into gastrocnemius and 20 μl into tibialis anterior muscle. Mice were given 0.05–0.1 mg/kg buprenorphine 30 min prior to the procedure, and additional analgesia was administered after the procedure when necessary.

### 2.5 Exercise performance test

To measure exercise capacity, mice were first acclimatized to treadmill running for 3 consecutive days. On the first day, the mice ran for 10 min at a speed of 6 m/min, followed by 5 min at 6 m/min and 5 min at 9 m/min on the second day, and 5 min at 6 m/min, 2 min at 9 m/min and 2 min at 12 m/min on the third day. On the day of the exercise performance test, mice started running at 6 m/min and the speed was increased by 3 m/min every 3 min. Exhaustion was determined as the point where mice were no longer able to run on the treadmill despite the researcher gently prodding it by hand for 10 s, at which point the total running distance was recorded. The slope of the treadmill was set to a 10° upward incline.

### 2.6 Acute exercise

Mice were first subjected to the treadmill exercise performance test as described above. After a rest of at least a week, they were subjected to 60 min of treadmill running at 60% of the maximal speed reached during the exercise capacity test. The slope of the treadmill was set to a 10° upward incline. Control mice were acclimatized but not subjected to the acute bout of exercise.

### 2.7 Acute exercise (downhill)

Mice were acclimatized to treadmill running as described above. The following day, mice were subjected to treadmill running, consisting of 5 min at 6 m/min, followed by 55 min at 17 m/min. The slope of the treadmill was set to a 15° downward incline. Control mice were acclimatized but not subjected to the acute bout of exercise.

### 2.8 Exercise training

Mice were single-housed in cages with free access to a running wheel for 8 weeks (Tecniplast Activity Cage Systems, Starr Life Sciences Corp.). The wheel was locked 24 h before the mice were terminated for tissue collection. Sedentary controls were single-housed in cages with identical enrichment, except for the absence of a running wheel, for the same duration.

### 2.9 Body composition (DEXA)

Mice were anaesthetized using isoflurane and their body composition was measured by dual-energy X-ray absorptiometry (DEXA) using a Lunar PIXImus2 or a Medikors InAlyzer for the Mustn1 KO-first or KO mouse, respectively.

### 2.10 Intraperitoneal glucose tolerance test

Mice were fasted for 5 h, weighed and 2 g/kg glucose was injected intraperitoneally. Blood glucose levels were measured at baseline and 15, 30, 60, 90 and 120 min after injection using a Freestyle Precision Neo (Abbott Laboratories) blood glucose meter.

### 2.11 Tail-cuff measurement of blood pressure

Blood pressure was measured using a CODA (Kent Scientific) tail-cuff system. Mice were placed in the animal holder, fitted the VPR and occlusion cuffs, and placed atop the warming platform (setting L2). On the first day, mice were acclimatized to the procedure without measurement. With at least 2 days of interval, measurements were then performed on 3 occasions and averaged.

### 2.12 Femoral artery ligation

Mice were anaesthetized using isofluorane, the proximal and distal portions of the left femoral artery were ligated and arteriectomy was performed between these two sites. The incision was then sutured, and mice left to recover on a heating pad. Mice were given analgesia (Carprofen/Rimadyl, 100 μl at 1 mg/ml) after the procedure and for the first 3 days post-surgery.

### 2.13 Force measurement in skeletal muscle

Force measurements were performed *ex vivo* on intact skeletal muscles, as previously described [21]. Briefly, mice were euthanized, and soleus and extensor digitorum longus (EDL) muscles were immediately excised, mounted on a force transducer (World Precision Instruments) and stimulated to measure the force-frequency relationships, fatigue resistance and recovery from the fatigue protocol.

### 2.14 Force measurement in aortic rings

Force measurements were performed *ex vivo* on aortic rings, as previously described [22]. Briefly, mice were euthanized, and the descending thoracic aorta was dissected and cut into aortic rings. Aortic rings were then mounted on a multi wire myograph system (Model 620 M; Danish Myo Technology, Denmark) and the reactivity of the vascular smooth muscle cells was measured using a solution containing 120 mM KCl (KPSS). Dose-dependent contraction with phenylephrine (PE), and subsequent dose-dependent relaxation with acetylcholine (ACh; endothelium-dependent) and sodium nitroprusside (SNP; endothelium-independent) were measured.

### 2.15 Cloning

The coding region of mouse Mustn1 (NM_181390.3) and an mRNA sequence containing 5’ and 3’ untranslated regions (AK003945.1) were amplified from muscle RNA using Phusion High-Fidelity DNA Polymerase (New England Biolabs). The resulting PCR products were ligated into intermediate vector pJET1.2/blunt (Thermo Fisher Scientific). The CDS was then cloned into expression vector pFLAG-CMV-5a (Sigma–Aldrich; contains a C-terminal FLAG-tag) and together with a N-terminal FLAG-tag into expression vector pcDNA3.1 (Invitrogen). The mRNA sequence (untagged) was cloned into expression vector pCMV5 (Dr. David Russell, UTSW). The sequences of all constructs were verified.

### 2.16 Generation of recombinant adenoviruses

To generate recombinant adenovirus, the AdEasy vector system (Agilent Technologies) was used according to the manufacturer's instructions. Briefly, the Mustn1 mRNA sequence as described above was cloned into the intermediate pAdTrack-CMV vector (which expresses a GFP tracer), followed by *in vivo* recombination into virus production vector pAdEasy-1. Ad-293 cells were then used for virus production and amplification. Control adenovirus was generated in the same way using an empty pAdTrack-CMV vector (also expresses the GFP tracer).

### 2.17 Cell culture, transfection, and immunocytochemistry

293T (ATCC ref. CRL-3216) and Ad-293 (Agilent Technologies ref. 240,085) cells were grown in DMEM supplemented with 10% fetal bovine serum and 100 U/ml penicillin/streptomycin. For immunofluorescence experiments, 293T cells were grown on collagen-coated coverslips and transfected with 1 μg/ml plasmid DNA (expressing N- or C-terminally Flag-tagged, or untagged Mustn1) using 5 μg/ml polyethylenimine (PEI MAX, Polysciences ref. 24,765). 24 h after transfection, cells were fixed with 4% paraformaldehyde in 0.1M phosphate buffer (PBS) for 15 min, washed three times with PBS, permeabilized for 15 min (0.25% Triton X-100 in PBS), and incubated with blocking solution (10% goat normal serum in PBS) for 15 min, all at room temperature. Cells were then incubated with rabbit anti-Mustn1 antibody (Merck Millipore, ABD115, 1:500) overnight at 4 °C, washed three times with PBS, followed by incubation with AlexaFluor 594 goat anti-rabbit antibody (Invitrogen, A11037, 1:500) for 1 h at room temperature. Both primary and fluorophore-conjugated secondary antibodies were diluted in blocking solution. Cells were washed three times with PBS, counter-stained with DAPI and mounted on a microscopy slide using Vectashield for imaging. Images were captured using a Zeiss Axio Vert.A1 widefield fluorescence microscope with a LD Plan-Neofluar 40x/0.6 Korr M27 objective.

### 2.18 Smooth muscle cell isolation and culture

Descending thoracic aortae from 3 individual Mustn1-KO mice or WT littermates at 4–8 weeks of age were harvested and cleaned of periadventitial fat. Isolated aortae were enzymatically digested in Hank's balanced salt solution (HBSS) containing 1 mg/ml collagenase type 2, 1 mg/ml soybean trypsin inhibitor, and 0.744 U/ml elastase (all from Worthington Biochemical) for 10 min at 37 °C in a humidified 5 % CO_2_ incubator. The adventitia was then stripped away, and endothelial cells were gently scraped off the luminal surface. The 3 aortae were then pooled and cut into 1 mm pieces and further digested enzymatically for an hour. Dissociated SMCs were then cultured in DMEM/F-12 containing 20% heat-inactivated FBS and 100 U/ml penicillin-streptomycin. After passage 3, cells were cultured in 10% heat-inactivated serum-containing media. This protocol was adapted from Dr. Gary Owens Lab (University of Virginia).

### 2.19 Smooth muscle cell secretion assay

Primary mouse smooth muscle cells were transduced with an adenovirus for ectopic expression of Mustang or a control adenovirus (MOI 200). 2 days post-transduction, cells were washed twice with culture medium (without fetal bovine serum). 3, 6, and 9 h after washing, cell supernatant was collected, and cells were lysed for western blotting. Proteins from the cell supernatant were precipitated with 0.15 vol. trichloroacetic acid overnight. Protein pellet was resuspended in Laemmli buffer, and denatured at 65 °C for 30 min.

### 2.20 Western blotting

Protein was extracted from cells using RIPA buffer (50 mM tris pH 7.5, 150 mM sodium chloride, 1 mM EDTA, 20 mM glycerol-2-phosphate, 5 mM sodium pyrophosphate, 50 mM sodium fluoride, 0.5 mM PMSF, 1 mM dithiothreitol, 1 mM sodium orthovanadate, 1% (w/v) Triton X-100, 0.5% (w/v) sodium deoxycholate, 0.1% (w/v) sodium dodecyl sulfate). Lysates were cleared at 13,000 g for 15 min at 4 °C and denatured in Laemmli buffer (50 mM tris pH 6.8, 2% (w/v) sodium dodecyl sulfate, 1 mM EDTA, 6.25% (v/v) glycerol, 0.01% (w/v) bromophenol blue, 100 mM dithiothreitol). Samples were then separated by SDS-PAGE and transferred to PVDF membrane. Membranes were blocked for 1 h in 20 mM tris (pH 7.6), 137 mM NaCl, 0.1% (v/v) Tween-20 (TBST) containing 5% (w/v) skimmed milk. Membranes were incubated in primary antibody prepared in TBST containing 1% (w/v) BSA overnight at 4 °C. Detection was performed using horseradish peroxidase-conjugated secondary antibodies and enhanced chemiluminescence reagent. Primary antibodies used were against Mustn1 (Merck Millipore, ABD115, 1:1000), Akt (Cell Signaling Technology, #9272, 1:1000) and α-tubulin (Sigma–Aldrich, T6199, 1:2000). Protein was quantified by densitometry using “Volume Tools” in Image Lab (Version 6.0.1. build 34, Biorad Laboratories Inc.).

### 2.21 Smooth muscle cell proliferation and migration assays

Smooth muscle cells isolated from mouse aorta were subjected to proliferation and migration assays as previously described [23]. Briefly, experiments were performed using the IncuCyte S3 Live-Cell Analysis System (Sartorius). For the proliferation assay, cells were plated at 60–70% confluence on a regular 48-well plate, and images were taken every 2 h for 96 h. For the migration assay (wound-healing assay), cells were plated at 100% confluence on an IncuCyte ImageLock 96-well plate (Sartorius), and a homogeneous 700–800 μm wide wound was created using the IncuCyte WoundMaker tool (Sartorius). Images were taken every 2 h for 60 h. For both assays, images were analyzed using IncuCyte Base software with the Scratch Wound Analysis Software Module (Sartorius). Experiments were repeated with smooth muscle cells originating from 3 independent isolations for each genotype.

### 2.22 Tissue collection

All tissues were collected, immediately frozen in liquid nitrogen, and stored at −80 °C until analysis. Gastrocnemius muscle and heart were powdered in a dry ice-cooled Bessman pulverizer before further processing.

### 2.23 RNA extraction, cDNA synthesis and quantitative PCR

Total RNA was extracted from cells and frozen tissue using TRI reagent (Sigma–Aldrich ref. T9424) according to the manufacturer's instructions. Purified RNA was then treated with DNAse I (Invitrogen, 18068015) and used to prepare cDNA using the High-Capacity RNA-to-cDNA Kit (Applied Biosystems, 4387406). Quantitative PCR was conducted on a QuantStudio 6 Flex Real-Time PCR system (Applied Biosystems) using SYBR Green PCR Master Mix (Applied Biosystems, 4368708), with primers as reported in Supplementary File 1. Ct values were used to calculate gene expression, expressed either as relative expression to a house-keeping gene as indicated (2^-Δ^^Ct^) or fold change (2^-ΔΔ^^Ct^) normalized to the experimental condition as indicated and using one or the geometric mean of several housekeeping genes as appropriate.

### 2.24 Muscle cross-sections and immunohistochemistry

Tibialis anterior muscle was dissected, mounted on a piece of cork using gum tragacanth and frozen in liquid nitrogen-cooled 2-methylbutane. Muscle cross-sections of 10 μm thickness were cut using a cryostat and transferred to a microscopy slide. Sections were fixed with 4% paraformaldehyde in 0.1M phosphate buffer (PBS) for 12 min, washed two times with PBS, and incubated with blocking solution (3% fetal bovine serum/0.1% Triton X-100 in PBS) at room temperature for 1 h. Sections were then incubated with primary antibodies overnight at 4 °C followed by incubation with fluorophore-conjugated secondary antibodies for 1 h at room temperature. Both primary and fluorophore-conjugated secondary antibodies were diluted in blocking solution. Primary antibodies used were rabbit anti-Mustn1 (Merck Millipore, ABD115, 1:500) and mouse anti-α-actin (α-SMA) AlexaFluor 647 (Santa Cruz, sc-32251, 1:100). Secondary antibody AlexaFluor 546 donkey anti-rabbit (Invitrogen, A10040, 1:1000) was used to detect Mustn1 primary antibody. Sections were washed three times with PBS, mounted using Vectashield and coverslipped for imaging. Images were captured using a Zeiss Axio Observer LSM 700 confocal microscope with a Plan-Apochromat 20x/0.8 M27 objective. Images were processed using ZEN lite (version 3.8.99.01000, Carl Zeiss Microscopy GmbH). Intensity thresholds for each marker were adjusted equally across images. For Mustn1 signal, the lower intensity signal was adjusted using the Mustn1-KO tissue as a reference.

### 2.25 Collagen content

Collagen content was calculated from hydroxyproline content measured by a colorimetric assay based on hydroxyproline oxidation to a pyrrole intermediate followed by reaction with Ehrlich's reagent to produce a chromophore, adapted from a previously described protocol [24]. Briefly, tibialis anterior muscles were dissected, with the tendon cut in a consistent way. Muscles were then accurately weighed and hydrolyzed with 5 M NaOH (100 °C for 16 h, shaking at 1200 rpm), neutralized with HCl and clarified at 10,000 g for 5 min. Samples were then oxidized in the presence of 50 mM chloramine T (56 mM stock dissolved in 10% (v/v) ddH_2_O, 10% (v/v) isopropanol, 80% (v/v) citrate-acetate buffer pH 6 (50 g citric acid monohydrate, 12 ml glacial acetic acid, 72.3 g sodium acetate anhydrous, 34 g sodium hydroxide in 1L)) for 25 min at room temperature. Samples were then reacted with 0.5 M DMAB (Ehrlich's reagent, 1 M DMAB stock dissolved in 70% (v/v) isopropanol, 30% (v/v) hydrochloric acid) for 20 min at 65 °C. Reaction was quenched by placing the samples on ice, and absorbance at 550 nm was measured on a spectrophotometer.

### 2.26 RNA sequencing

RNA was extracted from frozen tissue as described above. RNA (1 μg) was further purified using a Direct-zol RNA Miniprep kit (Zymo Research) including on-column DNAse I treatment. RNA quality was assessed using a 2100 Bioanalyzer system (Agilent Technologies) with an RNA integrity number (RIN) greater than 8 as a threshold. Random primed cDNA library preparation with polyA-enrichment and Illumina sequencing (single reads, 50 bp, 30M reads per sample) were performed by Eurofins Genomics.

### 2.27 RNA-seq analysis

Reads were aligned to the GRCm38.p6 mouse genome using STAR [25]. Feature count was performed using featureCounts [26], and differential expression analysis using DESeq2 [27] with adaptive log-fold change shrinkage estimator [28]. Differential gene expression threshold was set to adjusted p-value <0.05.

### 2.28 Aorta and skeletal muscle proteomics

Tibialis anterior and descending thoracic aorta were collected as described above, individually pulverized in liquid nitrogen, and resuspended in about 5 volumes of LYSE buffer (PreOmics), heated at 95 °C for 5 min under continuous shaking and sonicated in a water-bath sonicator (Diagenode) for 15 min with a 50% duty cycle. Protein concentration in the lysate was measured with a fluorometric method based on a tryptophan standard curve [29] and subsequently adjusted to 2 μg/μl. Proteolytic digestion was carried out by adding 1 μg of endoproteinase LysC and trypsin per 50 μg of lysate. After overnight digestion at 37 °C under continuous shaking, the lysate was acidified to a final concentration of 0.1% trifluoro-acetic acid (TFA) and loaded onto StageTip plugs of SDB-RPS. Purified peptides were eluted with 80% acetonitrile - 1% ammonia and dried. Tibialis anterior samples were analyzed in technical triplicates.

#### 2.28.1 Mass spectrometry data acquisition

Peptides were separated on 50 cm columns (75 μm inner diameter) of ReproSil-Pur C18-AQ 1.9 μm resin (Dr. Maisch GmbH) packed in-house. The columns were kept at 60 °C using a column oven. Liquid chromatography was carried out on an anEASY-nLC-1200 system coupled through a nanoelectrospray source to an Orbitrap Exploris mass spectrometer (Thermo Fisher Scientific). Peptides were loaded in buffer A applying a nonlinear 120-min gradient of 0–65% buffer B at a flow rate of 300 nl/min. MS data were collected in data-independent acquisition (DIA) mode, consisting of one MS1 full scan (300–1,650 *m*/*z* range) acquired at a resolution of 120,000 at 200 *m*/*z* with normalized automatic gain control (AGC) target set to 300 and 78 MS2 scans collected at a resolution of 15,000, an isolation window of 17.3 *m*/*z* and window overlap of 1. Cycle time was 3 s. HCD collision energy was set at 30%.

#### 2.28.2 Mass spectrometry data analysis

DIA raw files were processed using DIA-NN, version 1.8.1 [30] using FASTA digest for library-free search and deep learning-based spectra and retention time prediction. Missed cleavages were set to 1, N-term N excision and cysteine carbamylation were set as fixed modifications. Match between runs was enabled. Tibialis anterior and aorta samples were quantified in separate sessions.

Downstream analysis was performed with Perseus, version 2.0.10.0 [31]. For significance analysis, Student's t-tests were performed with s0 set to 0.1 and with significance threshold set at 5% using permutation-based FDR with 250 randomizations. When stated, missing values were imputed assuming a normal distribution with a width of 0.3 and with a downshift of 1.8, separately for each sample.

### 2.29 Muscle extracellular fluid proteomics

Extracellular fluid (EF) proteomics was adapted from a previously described protocol [32]. Quadriceps muscle was dissected and placed into a nylon net filter with 20 μm pore size (Millipore, NY2004700) and fixed in a 1.5 ml tube. Tissues were centrifuged at 1,000 g for 10 min at 4 °C and the extracted fluid stored at −80 °C until further processing. Quadriceps EF of two mice were combined to a total of 4 samples per condition. EF was diluted 1:1 with PBS and centrifuged at 3,200 g for 10 min at 4 °C. Supernatant was transferred to an ultracentrifugation tube and centrifuged at 100,000 g at 4 °C for 1 h to deplete extracellular vesicles. Supernatant was immunodepleted as previously described [32].

#### 2.29.1 Protein digest and peptide isobaric labeling

25 μg of immunodepleted EF were mixed 1:1 with protein lysis buffer (50 mM HEPES pH 8.8, 2% SDS, 150 mM NaCl, 5 mM dithiothreitol (DTT), Phosphatase Inhibitor (Sigma Aldrich, 04906837001), Protease Inhibitor (Sigma Aldrich, 11836170001)) and vortexed for 1 min. Samples were processed as previously described [32] with the following modifications: After reduction and alkylation, proteins were precipitated using methanol chloroform precipitation. Briefly, 3 parts of HPLC grade methanol, 1 part HPLC grade chloroform, and 2.5 parts HPLC grade water were added to each sample. Samples were vortexed and centrifuged at 4,000 g for 10 min. Supernatant was carefully removed to keep the protein disc in the interphase intact. Protein disc was washed three times with HPLC grade methanol at 4,000 g for 10 min. Protein pellets were dried and resuspended in 200 mM EPPS buffer (Fisher Scientific, J61476). Protein digestion and TMT labeling were performed as previously described [32]. Peptides were fractionated using high pH reversed-phase peptide fractionation kit (Pierce, #84868) according to the manufacturer's instructions. In total 8 fractions were collected. The fractions were dried in a vacuum centrifuge, resuspended in 3% acetonitrile, 0.5% formic acid and desalted by stage-tip. Peptides were eluted in 70% acetonitrile, 1% formic acid, dried, and resuspended in 5% acetonitrile, 5% formic acid. All fractions were analyzed by LC-MS/MS.

#### 2.29.2 Mass spectrometry data acquisition

All data were collected on an Orbitrap Eclipse mass spectrometer (Thermo Fisher Scientific) coupled to a Proxeon EASY-nLC 1000 LC pump (Thermo Fisher Scientific). Peptides were separated using a 90-min gradient at 500 nl/min on a 30-cm column (i.d. 100 mm, Accucore, 2.6 mm, 150 Å) packed inhouse. Data were collected as follows: High-field asymmetric-waveform ion mobility spectroscopy (FAIMS) was enabled during data acquisition with compensation voltages (CVs) set as 40 V, 60 V, and 80 V [33]. MS1 data were collected using the Orbitrap (60,000 resolution; maximum injection time 50 ms; AGC 4 3 105). Determined charge states between 2 and 6 were required for sequencing, and a 60 s dynamic exclusion window was used. Data dependent mode was set as cycle time (1 s). MS2 scans were performed in the Orbitrap with HCD fragmentation (isolation window 0.5 Da; 50,000 resolution; NCE 36%; maximum injection time 86 ms; AGC 1 3 105).

#### 2.29.3 Mass spectrometry data analysis

Raw files were first converted to mzXML, and monoisotopic peaks were re-assigned using Monocle [34]. Database searching included all mouse entries from Uniprot (downloaded in January, 2022). The database was concatenated with one composed of all protein sequences in the reversed order. Sequences of common contaminant proteins (e.g., trypsin, keratins, etc.) were appended as well. Searches were performed using the comet search algorithm. Searches were performed using a 50-ppm precursor ion tolerance and 0.02 Da product ion tolerance. TMTpro on lysine residues and peptide N termini (+304.2071 Da) and carbamidomethylation of cysteine residues (+57.0215 Da) were set as static modifications, while oxidation of methionine residues (+15.9949 Da) was set as a variable modification. Peptide-spectrum matches (PSMs) were adjusted to a 1% false discovery rate (FDR) [35]. PSM filtering was performed using linear discriminant analysis (LDA) as described previously [36], while considering the following parameters: comet log expect, different sequence delta comet log expect (percent difference between the first hit and the next hit with a different peptide sequence), missed cleavages, peptide length, charge state, precursor mass accuracy, and fraction of ions matched. Each run was filtered separately. Protein-level FDR was subsequently estimated at a data set level. For each protein across all samples, the posterior probabilities reported by the LDA model for each peptide were multiplied to give a protein-level probability estimate. Using the Picked FDR method [37], proteins were filtered to the target 1% FDR level. For reporter ion quantification, a 0.003 Da window around the theoretical *m*/*z* of each reporter ion was scanned, and the most intense *m*/*z* was used. Reporter ion intensities were adjusted to correct for the isotopic impurities of the different TMTpro reagents according to manufacturer specifications. Peptides were filtered to include only those with a summed signal-to-noise (S/N) of 160 or greater across all channels. For each protein, the filtered peptide TMTpro S/N values were summed to generate protein quantification.

#### 2.29.4 Western blot of extracellular fluid samples at different speeds

EF was isolated from quadriceps of wildtype mice using the indicated centrifugation speeds. EF volume was measured, and protein concentration was determined using bicinchoninic acid assay (Fisher Scientific, 23,225). Samples were denatured in LDS sample buffer (4X) (Invitrogen, NP0007) for 5 min at 95 °C. 10 μg of protein was resolved on a 4–12% NuPAGE BisTris SDS–PAGE (Invitrogen) with MOPS SDS Running Buffer (Sigma Aldrich, M1254), transferred to a polyvinylidene difluoride (PVDF) membrane (Millipore, IPVH00010) using transfer buffer (25 mM Tris, 192 mM glycine, 20% (v/v) methanol). To control for correct loading and transfer, Ponceau staining was performed according to manufacturer's instructions (Sigma Aldrich, P7170). Membranes were blocked in 5% BSA or 5% milk in TBS containing 0.05% Tween-20 (TBST) and incubated overnight at 4 °C with primary antibody. Secondary HRP-conjugated antibodies were used, membranes were incubated in Immobilon Crescendo Western HRP substrate (Fisher Scientific, WBLUR0500), and imaged (GE Amersham Imager AI680). The following antibodies were used: Mouse Monoclonal Anti-OXPHOS cocktail, Abcam Cat#: ab110413; RRID: AB_2629281; Rabbit Monoclonal Anti-GOLGIN 97, Cell signaling Cat#: 13,192; RRID: AB_2798144; Rabbit Monoclonal Anti-LAMIN A/C, Abcam Cat#: ab169532.

### 2.30 Gene ontology term overrepresentation analysis

Overrepresentation analysis was performed using g:Profiler [38]. Archive of the query and results for the RNA-seq data (biit.cs.ut.ee/gplink/l/_fFeAsQFSv) and the mass spectrometry data (biit.cs.ut.ee/gplink/l/8XqBcb3vS_) can be found on the g:Profiler website.

### 2.31 Publicly available datasets

RNA sequencing data of mouse gastrocnemius muscle upon hindlimb unloading and reloading was deposited to the Gene Expression Omnibus (GEO) data repository with accession number GSE237099. Data for *Mustn1/MUSTN1* expression were taken from these publicly available datasets: Human unilateral lower limb suspension and active recovery (GEO, GSE211204) [39], Muscular dystrophy with myositis (mdm) in muscles from mice (GEO, GSE210263) [40], Single cell analysis of the normal mouse aorta (The Broad Institute Single Cell Portal, SCP289) [41], Large-scale integration of single-cell transcriptomic in mouse skeletal muscle regeneration (Dryad, https://doi.org/10.5061/dryad.t4b8gtj34) [42], MuscleDB by Laura D. Hughes, Karyn Esser, and Michael E. Hughes (GEO, GSE100505) [43], An RNASeq normal tissue atlas for mouse and rat (ArrayExpress, E-MTAB-6081) [44]. Further data were downloaded as follows: Meta-analysis of Skeletal Muscle Response to Exercise (v3.2207 data from metamex.eu on 27/04/2023) [45], The Genotype-Tissue Expression (GTEx) project (v8 data from genome-mysql.cse.ucsc.edu on 02/05/2023) [46].

### 2.32 Data and code availability

RNA sequencing data from Mustn1 KO-first mice was deposited to GEO (GSE250506). The mass spectrometry proteomics data have been deposited to the ProteomeXchange Consortium via the PRIDE [47] partner repository with the dataset identifier PXD046610 (skeletal muscle and aorta from Mustn1 KO mice) and PXD047169 (extracellular fluid from mouse quadriceps). All code used to analyze data is available on GitHub (https://github.com/ruaslab).

### 2.33 Statistical analysis

Statistical analyses were performed using GraphPad Prism (version 10.0.3) with tests, comparisons and significance thresholds as indicated in the corresponding figure legends.

## 3 Results

### 3.1 Muscle reloading after disuse increases expression of the microprotein-encoding gene *Mustn1*

**Figure 1:**
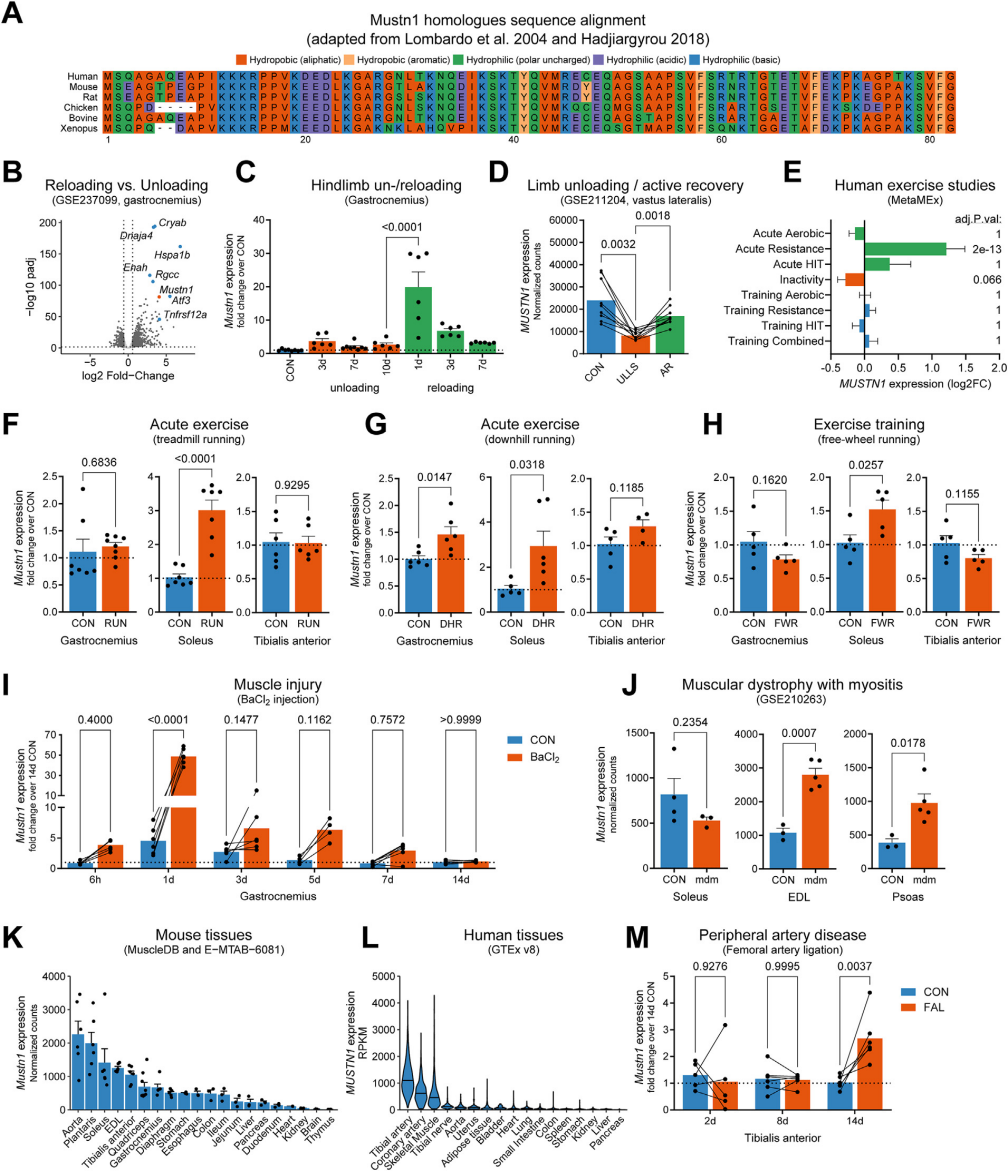
***Mustn1* is an exercise- and injury-responsive gene in skeletal muscle.** (A) Amino acid sequence alignment of Mustn1 homologues using the ggmsa R package. (B) RNA-seq analysis of gene expression in the gastrocnemius muscle of mice after 10 days of hindlimb unloading followed by 1 day of reloading, and controls (n = 2–4). Volcano plot shows genes differentially expressed between reloading day 1 and unloading day 10 (GSE237099). (C) qRT-PCR analysis of *Mustn1* gene expression in mouse gastrocnemius during hindlimb unloading and reloading. One-way ANOVA with Šidák correction for multiple comparison testing (unloading compared to controls, and reloading compared to 10 days unloading, n = 6–8), only p < 0.05 shown. (D) *MUSTN1* gene expression by RNA-seq in human muscle biopsies (vastus lateralis) collected from subjects at baseline (CON), after 10 days of unilateral limb suspension (ULLS), and after 21 days of active recovery (AR). Publicly available dataset (GSE211204). Repeated measures one-way ANOVA with Tukey correction for multiple comparison testing, only p < 0.05 shown. (E) Meta-analysis of *MUSTN1* gene expression from human exercise studies (MetaMEx). (F–H) qRT-PCR analysis of *Mustn1* expression in mouse muscle after different exercise protocols. Unpaired t tests. (F) Rested (CON) or 3 h after a bout of treadmill running on an upward slope (RUN) (n = 6–8). (G) Rested (CON) or 6 h after a bout of treadmill running on a downward slope (DHR) (n = 4–6). (H) Trained (FWR) and sedentary controls (CON) with or without access to a running wheel, respectively (n = 5). (I) qRT-PCR analysis of *Mustn1* gene expression in a mouse model of BaCl_2_-induced muscle injury of the gastrocnemius muscle (vs contralateral control limb). Repeated measures two-way ANOVA with Šidák correction for multiple comparison testing (n = 4–6). (J) *Mustn1* gene expression in skeletal muscles of mice with muscular dystrophy with myositis (mdm) compared with wild-type controls (CON). Publicly available dataset (GSE210263). Unpaired t test (n = 3–5). (K) *Mustn1* gene expression in mouse tissues from publicly available RNA-seq data (MuscleDB and E-MTAB-6081). (L) RNA-seq analysis of *MUSTN1* gene expression in human tissues, presented as violin plots (GTEx v8). (M) qRT-PCR analysis of *Mustn1* gene expression in tibialis anterior muscle after femoral artery ligation. Repeated measures two-way ANOVA with Šidák correction for multiple comparison testing (n = 5–6). Data are presented as mean values and SEM unless otherwise stated.

To identify genes important for the remodeling of skeletal muscle necessary for the transition from atrophy to compensatory hypertrophy, we used a mouse model of hindlimb unloading/reloading [48]. This is a well-established model that causes unloading-induced muscle atrophy (ca. 30% reduction in gastrocnemius mass after 10 days of unloading), followed by compensatory tissue regeneration and hypertrophy, which requires a similar period of time to reach control levels. C57BL/6J mice were divided into control (normal in-cage ambulation), Unloading (10 days of hindlimb unloading), or Reloading (10 days of unloading followed by 1 day of return to normal cage activity). Gastrocnemius muscles from the different groups were then used for analysis of gene expression by RNA-sequencing (RNA-seq). When determining differential gene expression at the transition from unloading to reloading, we found *Mustn1* as one of the genes with the highest positive fold-change (Figure 1A,B). *Mustn1* encodes an evolutionarily conserved, 82 amino acid-long microprotein (Figure 1A) previously reported to be involved in skeletal muscle development, myoblast differentiation [14], and metabolism [18]. In the same comparison we found genes involved in protein folding (*Cryab*, *Hspa1b*), cytoskeleton reorganization (*Enah*), cell cycle regulation (*Rgcc*), and stress response (*Atf3*) (Figure 1B). To investigate the temporal kinetics of *Mustn1* gene expression, we performed a hindlimb unloading/reloading experiment in which we collected gastrocnemius muscles at 3, 7 and 10 days of unloading, as well as after 1, 3 and 7 days of reloading. While there was a trend towards increased *Mustn1* expression during unloading, the highest levels of *Mustn1* were observed at 1 day of reloading (19.4-fold or 7.5-fold when compared to control or 10 days of unloading, respectively). This increase proved to be transient as on day 3 this change in expression was down to 2.6-fold (vs. unloading), and further down to 1.2-fold on day 7 (vs. unloading) (Figure 1C). In humans, *MUSTN1* expression was lower in skeletal muscle from volunteers that underwent limb unloading, but also increased during the active recovery phase that followed (Figure 1D) [39]. Furthermore, querying the MetaMEx resource (Meta-analysis of Skeletal Muscle Response to Exercise) [45] we could determine that *MUSTN1* expression is highly induced in human skeletal muscle by resistance exercise and reduced with inactivity (Figure 1E).

### 3.2 Skeletal muscle *Mustn1* expression is increased upon exercise and muscle damage

Considering the increase in *MUSTN1* expression with resistance exercise (which is also associated with tissue remodeling and hypertrophy), we decided to check *Mustn1* expression in mouse muscle following different exercise modes and injury models. Among the tested samples, we could see *Mustn1* expression change in an exercise type- and muscle-specific manner. In soleus, *Mustn1* expression increased consistently with single bouts of acute exercise (both uphill and downhill treadmill running) and 8 weeks of exercise training (free-wheel running), whereas in the gastrocnemius only acute downhill running resulted in increased *Mustn1* expression (Figure 1F,G, and H). Downhill running involves eccentric muscle contractions and is associated with muscle damage similar to intense resistance exercise [49]. This increase in *Mustn1* is therefore also consistent with increased *MUSTN1* in humans during resistance exercise (Figure 1E). Interestingly, the largest increase in *Mustn1* expression (almost 50-fold over controls) was seen 1-day after inducing muscle injury in mice by BaCl_2_ injection (Figure 1I). Also in this model, the induction in *Mustn1* expression was transient, and progressively returned to baseline as tissue regeneration progressed (Figure 1I). A more modest increase in *Mustn1* expression could also be seen in EDL and psoas muscle (but not soleus) in a mouse model of muscular dystrophy (Figure 1J).

### 3.3 Arteries and skeletal muscle express the highest *Mustn1* levels, followed by the gastrointestinal tract

*Mustn1* has previously been shown to have high expression in mouse skeletal muscle [50], which we could indeed observe in a broad RNA-seq panel of mouse tissues (Figure 1K). However, we also observed that *Mustn1* expression levels in arteries of both mice and humans were as high or higher than in skeletal muscle, followed by tissues of the gastrointestinal tract (Figure 1K,L). These data strongly suggest that *Mustn1* is expressed in both smooth and skeletal muscle. Interestingly, while *Mustn1* expression was highest in mouse aorta, *MUSTN1* expression in human aorta was not as high as in peripheral arteries such as the tibial or coronary arteries (Figure 1K,L). With this in mind, we measured *Mustn1* expression in tibialis anterior after femoral artery ligation (FAL), which is a well-established model of peripheral artery disease. While *Mustn1* expression was unaffected by FAL after 2 and 8 days, expression was increased 14 days after the ligation (Figure 1M). While many genes upregulated at the earlier stages after FAL relate to angiogenesis and inflammation, later stages have been shown to involve genes more related to anti-inflammation and extracellular matrix remodeling [51].

### 3.4 Heterozygous *Mustn1* deletion causes gene expression changes related to extracellular matrix remodeling in skeletal muscle of young mice

**Figure 2:**
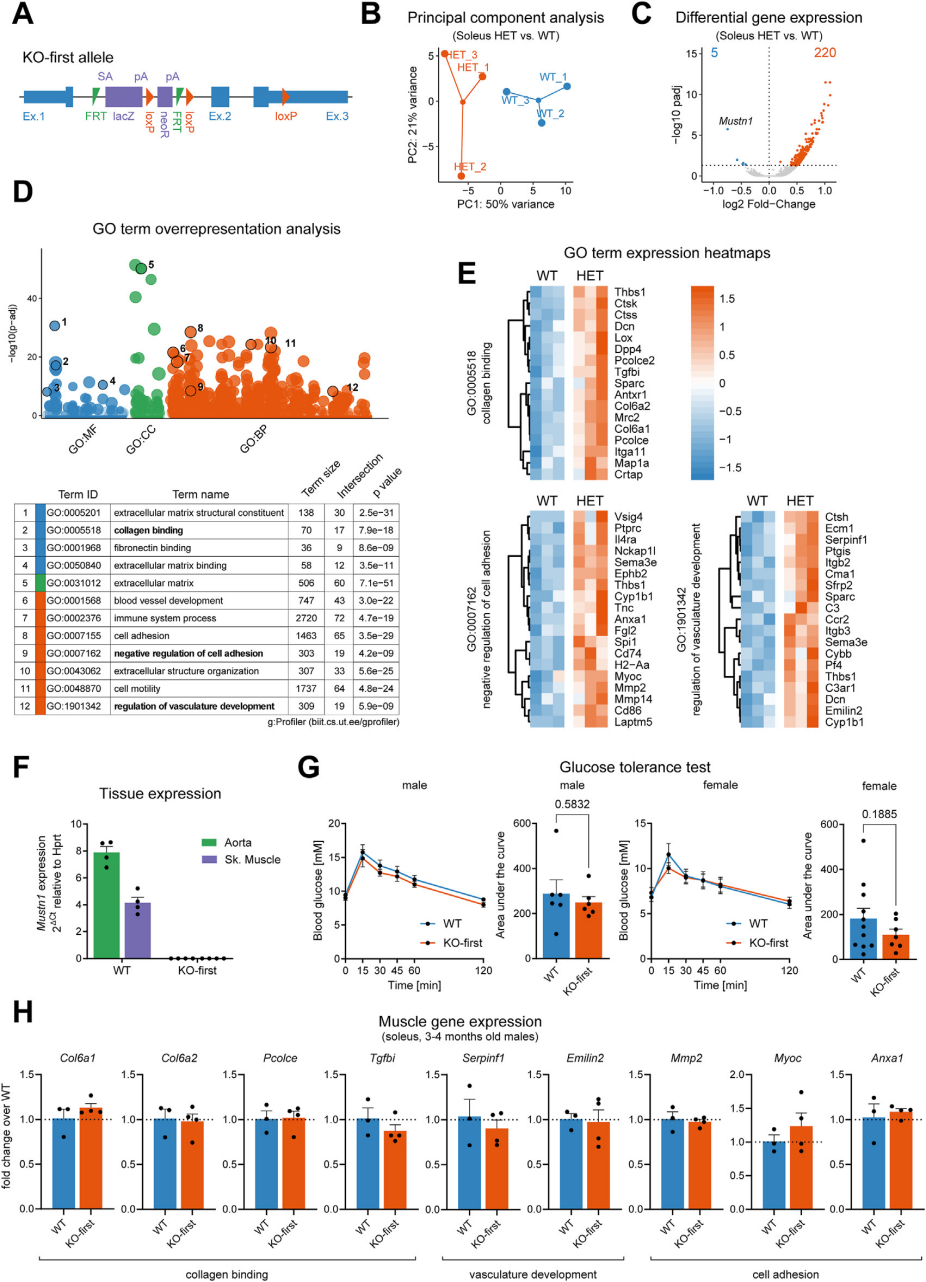
***Mustn1*-deficient mice (KO-first) show differences in muscle gene expression at young age but have normal muscle metabolism and function.** (A) Schematic representation of the Mustn1 KO-First (tm1a) allele obtained from the Mutant Mouse Resource & Research Centers (MMRRC). (B–E) RNA-seq analysis of gene expression in the soleus muscle from Mustn1 KO-First Heterozygous (HET) mice and wild-type (WT) littermate controls at 8 weeks of age. (B) Principal component analysis. (C) Volcano plot showing differentially expressed genes. (D) Gene Ontology (GO) term overrepresentation analysis using g:Profiler. (E) Gene expression heatmaps for selected GO pathways. (F) qRT-PCR analysis of *Mustn1* expression in descending thoracic aorta and skeletal muscle (tibialis anterior) of homozygous Mustn1 KO-First mice (n = 4). (G) Intraperitoneal glucose tolerance test (GTT) performed on 2-3-months-old male and female Mustn1 KO-first mice and WT littermate controls. Unpaired t tests (Area under the curve, n = 6 for males and n = 7–11 for females). (H) qRT-PCR analysis of genes identified in the RNA-seq shown in (E) using soleus muscle of homozygous Mustn1 KO-First mice and WT littermates at 3–4 months of age. Unpaired t tests (n = 3–4), no significant (p < 0.05) differences. Data are presented as mean values and SEM unless otherwise stated.

Due to the broader than expected *Mustn1* expression pattern, we obtained a whole-body “knockout first” mouse model (Mustn1 KO-first). The Mustn1 KO-first allele is designed to generate a null allele by splicing exon 1 of the targeted gene into a lacZ trapping element that contains a neomycin resistance gene (Figure 2A) [20]. We started by characterizing the heterozygous (HET) skeletal muscle (soleus) vs littermate wild-type (WT) controls from 8-week-old mice by RNA-seq (Figure 2B). Soleus muscle was chosen because of the consistent changes in *Mustn1* gene expression observed with exercise (Figure 1F,G and H). Differential gene expression analysis revealed very few genes with lower expression in HET vs. WT mice (one of which was *Mustn1,* as expected), and 220 with higher expression (Figure 2C and Supplementary File 2). Interestingly, gene ontology (GO) term overrepresentation analysis of the genes with higher expression in Mustn1 KO-first HET vs. WT revealed pathways related to extracellular matrix remodeling, vasculature development, and cell motility and adhesion (Figure 2D). Heatmaps for genes contributing to overrepresented GO terms (Figure 2E) are used to highlight some of the genes with known function or linked to disease. For example, genes contributing to the GO term “collagen binding” such as collagens *Col6a1* and *Col6a2* [52] and procollagen C-endopeptidase enhancer *Pcolce* (its paralog *Pcolce2* also figuring on the list) [53], have been linked to myopathies, while *Tgfbi* (transforming growth factor beta induced) has been linked to corneal dystrophies [54]. Contributors to the GO term “negative regulation of cell adhesion” on the other hand included matrix metalloproteinases *Mmp2* and *Mmp14* which can remodel the extracellular matrix, *Myoc* (myocilin) which encodes a secreted protein linked to glaucoma [55] and *Anxa1* (annexin A1), a mediator of glucocorticoid action [56]. Lastly, contributing to the GO term “regulation of vasculature development” are the anti-angiogenic serine (or cysteine) peptidase inhibitor, clade F, member 1 (*Serpinf1)* [57] and elastin microfibril interfacer 2 (*Emilin2)*, an extracellular matrix component conferring elasticity to blood vessels [58].

### 3.5 Loss of Mustn1 does not impair muscle function or whole-body glucose metabolism

Because previous studies have suggested Mustn1 play a role in myoblast differentiation [15] and whole-body glucose homeostasis [18], we decided to investigate muscle function and glucose homeostasis in homozygous Mustn1 KO-first mice. Homozygous Mustn1 KO-first mice were viable and born at the expected genotype and sex ratios (cf. Methods), and we saw no differences between male or female Mustn1 KO-first mice and WT controls in body weight (Supplementary Figure 1A) or body composition (Supplementary Figure 1B). To confirm the loss of *Mustn1* expression we tested aorta and skeletal muscle and, in both cases, could observe a >99.9% reduction in *Mustn1* transcript levels (Figure 2F). A recent study using a muscle-specific Mustn1 deletion mouse model (using Pax7-Cre-mediated gene deletion) reported that male (but not female) mice had improved glucose tolerance at 2 months of age, but no difference at 4 months of age [18]. We tested glucose tolerance in 2-3-months-old Mustn1 KO-first mice and WT littermates and saw no statistically significant differences between genotypes, although there was a trend towards improved glucose disposal in females (Figure 2G). We then further phenotyped the adult Mustn1 KO-first mice with regards to their muscle and aorta functions. No differences were observed in muscle-intrinsic properties using *ex vivo* muscle contraction assays (Supplementary Figure 1C), although female (but not male) Mustn1 KO-first mice had slightly higher exercise capacity than WT controls (Supplementary Figure 1D). We could not detect any differences in *ex vivo* aorta contraction tests or in blood pressure (Supplementary Figures 1E and F). Intrigued by these results, we wanted to check whether the previously observed changes in skeletal muscle gene expression in 8-week-old heterozygous KO-first mice persisted in the homozygous, 3–4 months old mice. To our surprise, none of the genes we tested showed any difference in expression between KO-first and WT (Figure 2H). Since this could be because rates of muscle growth and remodeling decrease with age, we decided to subject the Mustn1 KO-first mice to BaCl_2_-mediated muscle injury-regeneration. However, under these conditions, we detected an increase in *Mustn1* transcript in the contralateral, control leg of Mustn1 KO-first mice (Supplementary Figure 1G), presumably induced by the Rhabdomyolysis-like systemic effects of this intervention [59]. This shows that under certain conditions (such as pro-inflammatory stimuli), the artificial splice acceptor site used in the KO-first strategy to eliminate *Mustn1* expression is not 100% efficient, allowing for considerable endogenous gene expression, and rendering this model unusable as a KO.

### 3.6 Generation of a whole-body, Actb-Cre-driven Mustn1 KO mouse line

**Figure 3:**
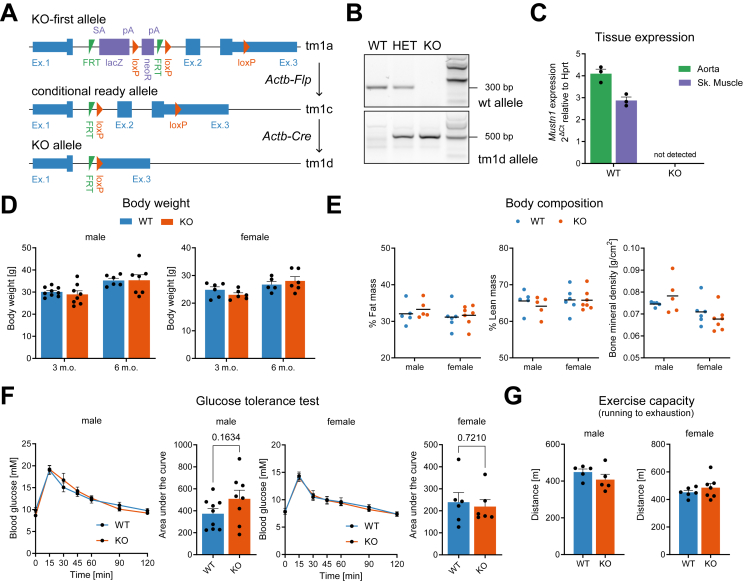
**Generation of a Actb-Cre driven Mustn1 KO mouse line** (A) Schematic representation of the different Mustn1 alleles to generate the whole body Mustn1 KO mouse line. (B) Genotyping result showing deletion of Mustn1 in mice with heterozygous (HET) and homozygous (KO) tm1a allele. (C) qRT-PCR analysis of *Mustn1* gene expression in descending thoracic aorta and skeletal muscle (tibialis anterior) of homozygous Mustn1 KO mice (n = 3). (D) Body weight of male and female Mustn1 KO mice and WT littermates at 3 and 6 months of age (n = 6–9 for males, n = 5–6 females). (E) Body composition of male and female Mustn1 KO mice and WT littermates at 3 months of age (n = 5 for males, n = 6–7 females). (F) Intraperitoneal glucose tolerance test (GTT) performed on 3-months-old male and female Mustn1 KO mice and WT littermate controls. Unpaired t tests (Area under the curve, n = 8–9 for males and n = 6 for females). (G) Exercise capacity test (treadmill running to exhaustion) performed on 3.5-months-old male and female Mustn1 KO mice and WT littermates. Unpaired t tests (n = 5 for males, n = 6–7 females), no significant (p < 0.05) differences. Data are presented as mean values and SEM.

Since the Mustn1 KO-first line proved unusable under certain conditions, we next generated a whole-body Mustn1 KO using the loxP gene targeting strategy which is built into the KO-first allele (Figure 3A). First, we removed the lacZ-NeoR cassette by crossing the Mustn1 KO-first with a Flp recombinase expressing mouse line. This generated the floxed allele line (Figure 3A), which was then crossed with an Actb-Cre deleter mouse to generate whole-body Mustn1 KO mice (Figure 3A,B), which were viable and born at the expected genotype and sex ratios (cf. Methods). With this approach we were able to eliminate *Mustn1* expression as verified by qRT-PCR in both aorta and skeletal muscle (Figure 3C). The Mustn1 KO line showed no differences in body weight and composition (Figure 3D,E), and no differences in glucose tolerance (Figure 3F). This confirms that in whole-body Mustn1 deficient mice (both KO and KO-first), glucose tolerance is not affected, and differences with previously reported data could be ascribed to slight differences in age, the fasting period (5-hour vs. 16-hour) or the genetic model used (Pax7-Cre-vs Myog-Cre-driven skeletal muscle Mustn1 deficiency) [18]. In contrast with the Mustn1 KO-first line, the Mustn1 KO showed no differences in exercise capacity when compared to littermate WT controls, even though mice in this experiment being older (3–4 vs. 2–3 months old) could be a confounding factor (Figure 3G).

### 3.7 *Mustn1* is expressed in vascular smooth muscle cells of the aorta, but Mustn1 deletion does not affect the aorta proteome

**Figure 4:**
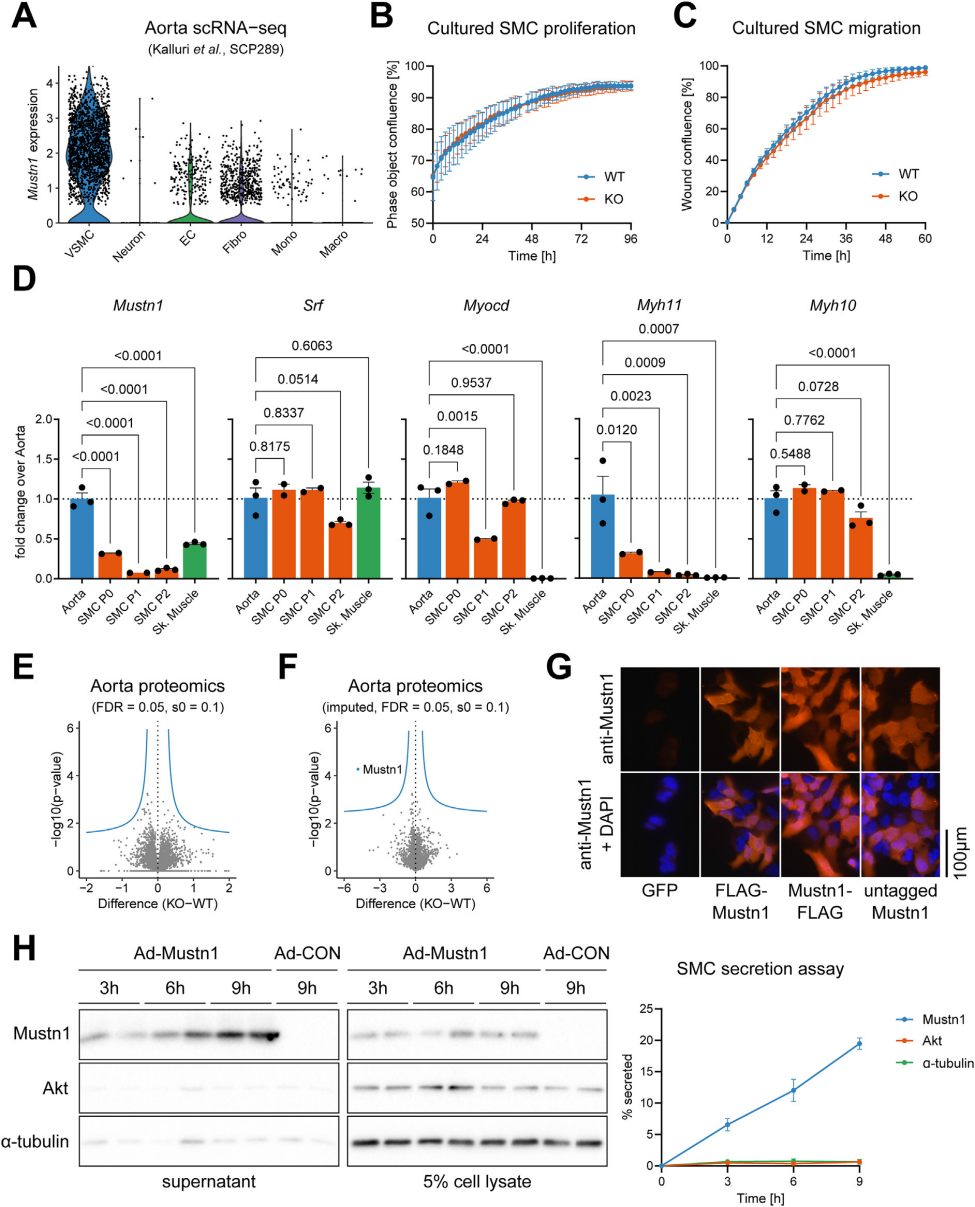
***Mustn1* is expressed in vascular smooth muscle cells of the aorta and is unconventionally secreted.** (A) Single cell RNA-seq analysis of mouse aorta. Violin plots showing *Mustn1* expression in different cell types (VSMC: vascular SMC, EC: endothelial cells, Fibro: fibroblasts, Mono: monocytes, Macro: macrophages). (B–C) Primary SMC isolated from aorta of Mustn1 KO and WT littermates were cultured and assayed for proliferation (B) and migration (C) (n = 3 independent experiments). (D) qRT-PCR analysis of gene expression in SMC isolated from aorta of C57BL/6N after initial plating (P0), at subsequent passages (P1, P2) as well as aorta and skeletal muscle tissues. One-way ANOVA with Dunnett correction for multiple comparison testing (only vs. Aorta, n = 2–3). (E–F) Mass spectrometry proteomics analysis of descending thoracic aorta from 3-month-old female Mustn1 KO and WT littermates. Volcano plots for non-imputed (E) and imputed (F) values (n = 4). Blue line represents the permutation-based FDR cut-off. (G) Immunofluorescence images of 293T cells stained using anti-Mustn1 antibody and DAPI shows ectopic Mustn1 (N- or C-terminal FLAG-tag, or untagged) localization. (H) Immunoblotting of cell supernatant and lysates of cultured SMC (isolated from C57BL/6N mice) transduced with Mustn1-expressing adenovirus (Ad-Mustn1) and control adenovirus (Ad-CON), collected 3, 6 or 9h after medium change. Representative blots (left panel) and quantification (right panel, n = 3). Data are presented as mean values and SEM unless otherwise stated. (For interpretation of the references to color in this figure legend, the reader is referred to the Web version of this article.)

Because of the high expression of *Mustn1* in mouse aorta (Figure 1K) we wondered if that could indicate high expression in smooth muscle cells (SMCs). Using publicly available scRNA-seq data from mouse aorta [41] we confirmed that vascular SMCs expressed by far the highest *Mustn1* levels, followed by discrete subpopulations of fibroblasts and endothelial cells (Figure 4A). To evaluate the cell-autonomous effect of Mustn1 deletion, we isolated SMCs from the descending thoracic aorta of Mustn1 KO and WT mice, evaluated their proliferation and migration capacity in culture, but saw no differences between genotypes (Figure 4B,C). Of note, qRT-PCR analysis of *Mustn1* expression in cultured SMCs showed that its expression levels drop considerably as soon as cells are placed in culture (Figure 4D), closely mimicking what happens to the expression of *Myh11*, the smooth muscle myosin heavy chain isoform [60]. This could of course have been an important factor when trying to detect differences between Mustn1 KO and WT SMCs in culture. Taking this into consideration we decided to perform a proteomics analysis of aortas from Mustn1 KO and WT mice, but this approach also didn't reveal any differences between genotypes, except for the expected reduction in Mustn1 levels in the KO animals (Figure 4E,F, and Supplementary File 3 and 4).

### 3.8 Mustn1 is secreted from smooth muscle cells

The subcellular localization of a GFP-Mustn1 fusion protein has been previously shown to be predominantly nuclear in MC3T3 pre-osteoblastic cells [50]. In line with this pattern of subcellular localization, Mustn1 has been postulated to be a transcriptional co-regulator [14,50,61]. However, taking together our data, including the lack of a cell-autonomous effect upon Mustn1 deletion (Figure 4B,C and E), we wondered if Mustn1 could have other, non-nuclear roles. To this end, we expressed different untagged or FLAG-tagged forms of Mustn1 in cultured cells followed by immunofluorescence imaging using an anti-Mustn1 antibody. Irrespective of the experimental condition (tagged or untagged Mustn1), we did not observe any exclusive nuclear localization, but rather a broad subcellular distribution including clear cytoplasmic localization (Figure 4G). In addition, bioinformatics analysis of the mouse and human Mustn1 protein identified it as a potential candidate for non-classical secretion (Outcyte score of 0.5495 and SecretomeP scores of 0.714 and 0.723 for mouse and human, respectively). Since these scores are only suggestive of potential protein secretion, we wanted to investigate this possibility experimentally. We then transduced cultured SMCs with an adenovirus for forced Mustn1 expression or a control adenovirus (both adenoviruses expressing GFP). While the levels of ectopic Mustn1 inside the cells remained constant over time, we could see a clear time-dependent accumulation of Mustn1 in the cell supernatant, with about 20% Mustn1 (compared to intracellular levels) secreted over the course of 9 h (Figure 4H). This was not observed for intracellular proteins such as Akt and α-tubulin (Figure 4H). Taken together, this data suggest that Mustn1 is a secreted microprotein.

### 3.9 Mustn1 protein is detected in muscle arterioles and secreted into the extracellular fluid upon hindlimb reloading

**Figure 5:**
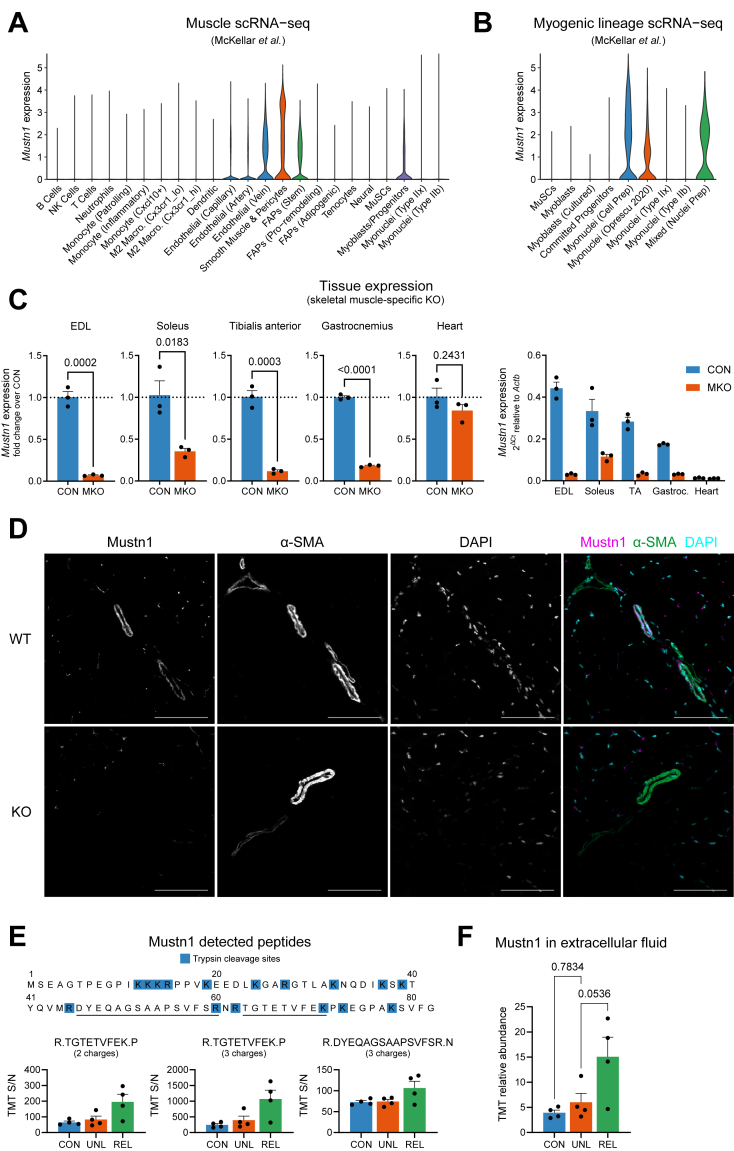
**Mustn1 protein is detected in muscle arterioles and secreted into the extracellular fluid upon hindlimb reloading.** (A) Integrated analysis of single cell and nuclei RNA-seq of mouse skeletal muscle. Violin plots showing *Mustn1* expression in different cell types. (B) Separate integration of (A) for myogenic cell types. (C) qRT-PCR analysis of *Mustn1* gene expression in different muscles from Mustn1 skeletal muscle-specific KO mice (MKO) and littermate controls. Unpaired t tests (n = 3). (D) Representative immunofluorescence images of tibialis anterior muscle cross-sections from Mustn1 KO mice and WT littermates, stained using Mustn1 and α-smooth muscle actin (α-SMA) antibodies, and DAPI, scale bars: 100 μm (n = 3). (E–F) Mass spectrometry identification of Mustn1 in mouse skeletal muscle (quadriceps) extracellular fluid under control, hindlimb unloading, or reloading conditions. Identified peptides and quantified raw tandem mass tag signal (TMT S/N) for each peptide (E) and total relative abundance of Mustn1 protein (F). One-way ANOVA with Dunnett correction for multiple comparison testing (only vs. UNL, n = 4). Data are presented as mean values and SEM unless otherwise stated.

After aorta, *Mustn1* expression is highest in mouse muscle and highly inducible with exercise and upon injury (Figure 1). Looking at a published resource that integrates muscle single-cell/nucleus RNA-seq datasets [42], we confirmed *Mustn1* expression in vascular SMCs, with further expression in venous endothelial cells and a subset of fibro-adipogenic progenitors (FAP), as well as in a cluster of cells labelled “Myoblasts/Progenitors” (Figure 5A). To better understand the latter, we also looked at the separate data integration provided for the cells of the myogenic lineage, with *Mustn1* expression mainly attributed to certain “Myonuclei” clusters and a “Mixed (Nuclei prep)” cluster (Figure 5B). These results show that it is difficult to compare expression levels when comparing single-cell and -nucleus transcriptomic data, which doesn't allow us to make a definitive statement about relative *Mustn1* transcript abundance between SMCs and skeletal muscle fibers. To remediate this problem at least at the whole-muscle level, we generated a skeletal muscle-specific knockout mouse by crossing our Mustn1 floxed-allele carrying mouse (Figure 3A) with a Mef2c enhancer/Myogenin promoter-driven Cre mouse line. We determined *Mustn1* expression in various muscle beds and in the heart and found that cell types other than skeletal muscle fibers contributed 7% (EDL), 35% (soleus), 12% (tibialis anterior) and 18% (gastrocnemius) to *Mustn1* expression (Figure 5C). This shows that particularly in a slow-oxidative muscle like soleus, and despite skeletal muscle fibers making up the bulk of muscle volume, non-fiber cell contribution to *Mustn1* expression is considerable. As expected, Myogenin-Cre driven recombination did not affect *Mustn1* expression in the heart (Figure 5C). More importantly, we were interested in knowing how Mustn1 protein expression was distributed within muscle. For this, we performed anti-Mustn1 immunostaining on tibialis anterior muscle cross-sections from WT and Mustn1 KO mice. We found strong and specific Mustn1 immunoreactivity to be localized to the larger vessels of the muscle, overlapping with staining for α-smooth muscle actin (α-SMA) (Figure 5D and Supplementary Figure 2). Interestingly, only α-SMA-positive vessels morphologically resembling arterioles rather than venules were also Mustn1-positive (Figure 5D).

To determine whether endogenous Mustn1 is secreted in skeletal muscle *in vivo*, we isolated interstitial/extracellular fluid from muscle using a recently published protocol [32], followed by quantification of Mustn1 by mass spectrometry (Supplementary File 5). Using muscle from C57BL/6N mice that underwent the hindlimb unloading/reloading protocol, we detected 3 Mustn1peptides, thus confirming that Mustn1 is indeed a secreted protein *in vivo* (Figure 5E). This result was further validated by determining the absence of known exclusively intracellular proteins such as components of the mitochondrial oxidative phosphorylation machinery, golgin, and lamin A/C (Supplementary Figure 3). Finally, by comparing the extracellular fluid isolated from muscle under the different conditions we found a trend towards increased Mustn1 protein abundance (3.8-fold, p = 0.0536) after 1 day of reloading (Figure 5F), mirroring its increase in gene expression (Figure 1B,C).

### 3.10 Muscles from Mustn1 knockout mice show changes in extracellular matrix composition and females display mild fibrosis after injury

**Figure 6:**
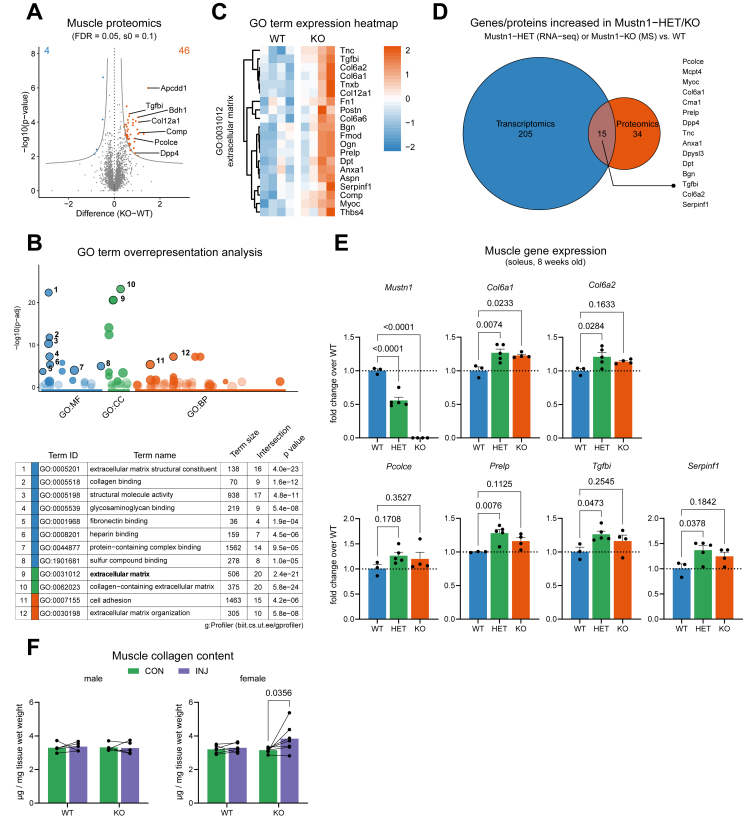
**Muscles from Mustn1 KO mice show changes in extracellular matrix composition and females display mild fibrosis after injury.** (A–D) Mass spectrometry proteomics analysis of tibialis anterior from 3-month-old female Mustn1 KO and WT littermates (n = 4). (A) Volcano plots showing differentially expressed proteins (grey line represents the permutation-based FDR cut-off). (B) Gene Ontology (GO) term overrepresentation analysis of proteins increased in the Mustn1 KO using g:Profiler. (C) Protein expression heatmap for the GO term “extracellular matrix”. (D) Venn diagram comparing differentially expressed proteins with differentially expressed genes in Mustn1 KO-first HETs vs. WT (Figure 2). (E) qRT-PCR analysis of gene expression in soleus muscle of 8-week-old homozygous (KO) and heterozygous (HET) Mustn1 KO mice, and WT littermates. One-way ANOVA with Dunnett correction for multiple comparison testing (only vs. WT, n = 3–5). (F) Muscle collagen content in tibialis anterior of 4-5-month-old male and female Mustn1 KO mice and WT littermates measured 14 days after cardiotoxin-induced muscle injury. Repeated measures two-way ANOVA with Šidák correction for multiple comparison testing (n = 5 for males, n = 6–7 for females). Data are presented as mean values and SEM unless otherwise stated.

To further characterize the Mustn1 KO mouse model (i.e., Actb-Cre-mediated, whole-body Mustn1 deletion) we next performed skeletal muscle proteomics. We found 4 proteins that were significantly lower and 46 proteins that were significantly higher in tibialis anterior muscle of Mustn1 KO compared to WT littermates (Figure 6A and Supplementary File 6). Among the most increased in the Mustn1 KO were proteins such as Apcdd1, which is a membrane-bound glycoprotein and an inhibitor of the Wnt signaling pathway [62], d-β-hydroxybutyrate dehydrogenase I (Bdh1), which is an enzyme involved in ketone body metabolism that has been shown to play a role in heart failure and fibrosis [63], Cartilage Oligomeric Matrix Protein (Comp), which has been shown to contribute to the contractile phenotype of vascular SMCs [64], and Dipeptidyl Peptidase 4 (Dpp4), which is a serine protease responsible for the degradation of incretins such as GLP-1 and thus plays a major role in glucose homeostasis, but which has also been shown to regulate blood flow in muscle [65] (Figure 6A). Strikingly, when we performed GO term overrepresentation analysis of the proteins with higher levels in Mustn1 KO, we found Molecular Function terms related to ECM component binding, Biological Process terms related to cell adhesion and ECM organization, and Cellular Component terms related to the ECM itself (Figure 6B). Looking at a heatmap of proteins contributing to the ECM term (Cellular Components), we found several collagens (Col6a1, Col6a2, Col6a6, Col12a1) and proteoglycans (Bgn, Fmod, Ogn, Aspn), amongst others (Figure 6C). Interestingly, comparing the RNA-seq results from heterozygous Mustn1 KO-first mice (Figure 2C) with the proteomics data from the Mustn1 KOs (each vs. their corresponding WT controls), showed a significant overlap of differentially expressed genes/proteins (Figure 6D). It is noteworthy that in this comparison, mouse model (heterozygous KO-first vs. homozygous KO), age (8-week-old vs. 3–4 months old), muscle type (soleus vs. tibialis anterior) and of course measurement (transcriptomics vs. proteomics) were all different. Nevertheless, in both instances, Mustn1 deficiency led to changes in cell adhesion and ECM organization. To check that gene expression changes observed by RNA-seq (Figure 2C) were also present in the Mustn1 KO mouse model, we quantified several transcripts in heterozygous and homozygous KO mice and found that they were indeed increased (Figure 6E). Lastly, to investigate the effect of Mustn1 deficiency in the context of muscle regeneration, we measured collagen content in muscle of Mustn1 KO mice and WT littermates after cardiotoxin-induced muscle injury. At 14 days after injury, no measurable fibrosis remained in WT mice when comparing the injured leg to the control leg. However, at the same time point of recovery from injury we could observe increased collagen content in female Mustn1 KO mice (but not in males; Figure 6F). Taken together, our data identifies Mustn1 as a microprotein secreted from SMCs that regulates ECM composition, with a potential role in skeletal muscle fibrosis.

## 4 Discussion

Microproteins are defined as proteins/peptides encoded by a small ORF of less than 100 amino acids, in contrast to other small proteins (such as many hormones) that are produced by proteolytic cleavage from longer (>100 amino acids) preproteins. The reason for making this distinction is that during the initial large-scale genome annotation efforts that followed the completion of the Human Genome Project, microproteins were often overlooked, as gene prediction algorithms were biased against small ORFs to reduce false positives [66]. More recently however, efforts to remediate this have shown that a large number of microproteins exist and that they can possess important biological activity [13]. For example, skeletal muscle-specific microproteins such as Myoregulin/Dworf [67,68] and Myomaker/Myomixer [69,70] have been shown to regulate calcium handling and myoblast fusion respectively. Another example is the downregulation of SPAR (Small regulatory polypeptide of amino acid response) upon muscle injury, which is important for muscle regeneration, highlighting the biological role and potential therapeutic relevance of microproteins [71]. Unlike these recently annotated microproteins, Mustn1 was first cloned in 2004 [50]. The Mustn1 ORF codes for a protein of 82 amino acids conserved in vertebrates [50]. To confirm that the ORF is indeed translated, here we show Mustn1 protein expression in the context of its native 5’-UTR. Furthermore, we report the mass spectrometric detection of endogenous Mustn1-derived peptides, unambiguously confirming its presence in mouse tissues.

Mustn1 has been described as a nuclear protein [50], due to the presence of basic amino acids towards its N-terminus which could act as a nuclear localization signal and the fact that GFP fusions of Mustn1 localized to the nucleus [50,72]. However, no studies to date have used and validated an antibody recognizing Mustn1, hence strong evidence of Mustn1 localization (endogenous or non-fusion) was lacking. Here, we used a polyclonal antibody raised against the C-terminus of Mustn1 (Millipore Cat. # ABD115) and showed that it can be used to detect Mustn1 by immunofluorescence, either ectopically expressed in cultured cells or the endogenous protein in the muscle microvasculature. Of note, while this antibody recognizes overexpressed Mustn1 by western blotting, we were not able to show a specific signal for endogenous Mustn1 using this technique. While we cannot exclude that in different contexts Mustn1 could be exclusively nuclear, under the conditions we tested it shows a broad subcellular distribution, which includes the nucleus and cytoplasm. Furthermore, we report that, despite the lack of a canonical signal peptide for endoplasmic reticulum sorting, Mustn1 can be found in the supernatant of cultured cells (Figure 4H), in muscle extracellular fluid (Figure 5E,F). In addition, we could also find Mustn1 in publicly available human plasma proteomics data [73]. This points towards unconventional protein secretion, which encompasses several possible mechanisms of secretion [74] and is thought to account for a substantial proportion of the secretome [75]. One example of this is basic fibroblast growth factor (bFGF/FGF-2), which has been shown to translocate across the plasma membrane [76]. While we do not know the mechanism by which Mustn1 is secreted, it has been suggested that biophysical properties of some microproteins could make them particularly amenable to unconventional protein secretion [77].

*Mustn1* is known to have high expression in adult mouse skeletal muscle [50,78], which was shown to increase during muscle regeneration [79]. Furthermore, it was reported that, in humans, eccentric muscle contractions increase *Mustn1* expression [80]. Here, we provide further evidence and an extensive overview of *Mustn1* expression with exercise, training, and muscle injury, both in mouse and human skeletal muscle.

Analysis of mouse and human tissues by bulk and single-cell RNA sequencing revealed *Mustn1* expression in SMCs, which had not been reported previously. In mouse muscle, we detected Mustn1 protein in SMCs of the muscle arterioles, in contrast to skeletal muscle fibers where the transcript was present, but protein could not be reliably detected by immunohistochemistry. Although we cannot exclude protein expression in other cell types including muscle fibers, SMCs appear to be the main source of Mustn1 protein. Therefore, and supported by our *in vitro* SMC secretion assays, it is likely that they are the source of secreted Mustn1 present in the muscle extracellular fluid. While it is well appreciated that skeletal muscle is an endocrine organ by secreting different peptides and proteins, collectively called myokines [2,81], whether some of those myokines originate from SMC is much less understood. Here, we propose the term “*leiokines*” for proteins such as Mustn1 that are secreted from SMCs. Whether secreted Mustn1 signals to other cells or whether it acts directly on, for example, components of the ECM remains to be investigated. Although the mechanism of action of SMC-secreted Mustn1 is not known, it is possible that a Mustn1 Receptor exists in target cells as is the case for cytokines (which include several microproteins). It is also possible that Mustn1 can remain associated with the ECM to, for example, modulate the activity of SMC receptors.

In this study, genetic loss of Mustn1 resulted in altered ECM components in mouse tibialis anterior muscle. It is interesting to note that we have observed differences in gene expression only in muscle of young adult (8-week-old) mice, but not older adult (3–4 months old) mice. Nevertheless, proteome analysis revealed the ECM to be altered in older adult Mustn1 KO mice. One possible explanation for this would be the implication of Mustn1 in muscle development, which bears many similarities with muscle regeneration after injury [3]. To get a first glimpse at a potential role of Mustn1 in muscle regeneration, we subjected Mustn1 KO mice to a muscle injury protocol and observed increased collagen content in female (but not male) mice 14 days after injury. This timepoint corresponds to the late stage of muscle regeneration [82], whereby in WT mice, fibrosis is resolved, and collagen content is not different in control and injured muscle. While Mustn1 does indeed seem to play a role in ECM dynamics during muscle regeneration, it would be interesting in the future to perform a time-resolved study to investigate levels of fibrosis at the different stages of muscle regeneration in Mustn1-deficient mice, and to measure the effect on muscle function. Considering some of the age-dependent observations in Mustn1 loss-of-function models, it is possible that there are compensatory mechanisms activated in the absence of Mustn1 expression, which deserve further investigation. Indeed, this idea is supported by recent studies showing changes in muscle fiber type composition in young Mustn1 KOs generated with a Pax7-Cre strategy [17]. In addition, aging studies using the Mustn1 models could reveal additional roles for this protein. Another interesting aspect related to the changes in ECM components is the significant overrepresentation of the GO term “cell adhesion” in both the transcriptomics and the proteomics data from Mustn1-KO mice. It is well known that the ECM and expression of cell adhesion molecules are crucial for the ability of immune cells to migrate and infiltrate tissues [83] which is crucial for muscle regeneration [84].

How the different cell types within skeletal muscle, in particular stromal cells such as FAPs, vascular SMCs, and pericytes (which all express *Mustn1* to varying degrees), participate in ECM remodelling and allow for immune infiltration, and how they signal between each other is only starting to be understood [85]. Fibrosis and inflammation are hallmarks of muscle injury and muscle dystrophies, and a better understanding of these processes could pave the way for more efficient therapies [86,87].

## Grants

This work was supported by grants from The 10.13039/501100004359Swedish Research Council and The 10.13039/501100009708Novo Nordisk Foundation to JLR (2016-00785 and NNF16OC0020804), from Stiftelsen Lars Hiertas minne and KI stiftelsen to SD, and from The 10.13039/501100004359Swedish Research Council, The Swedish Heart and Lung Foundation and The 10.13039/501100009708Novo Nordisk Foundation to MC (2020-01645, 20210431 and 2019–0055,026), and the National Institute of Health to 10.13039/100016838BMS (DK119117). SD received a postdoctoral fellowship from the Swiss National Science Foundation, JCC and IC from the Swedish Society for Medical Research, PRJ and BJ from the Wenner-Gren Foundations, and MJM from the German Research Foundation (Projektnummer 461079553). GOS was supported by the Erasmus+ programme of the 10.13039/501100000780European Union.

## CRediT authorship contribution statement

**Serge Ducommun:** Writing – review & editing, Writing – original draft, Visualization, Investigation, Funding acquisition, Conceptualization. **Paulo R. Jannig:** Writing – review & editing, Visualization, Investigation. **Igor Cervenka:** Writing – review & editing, Visualization, Investigation. **Marta Murgia:** Writing – review & editing, Investigation. **Melanie J. Mittenbühler:** Writing – review & editing, Investigation. **Ekaterina Chernogubova:** Writing – review & editing, Investigation. **José M. Dias:** Writing – review & editing, Investigation. **Baptiste Jude:** Writing – review & editing, Investigation. **Jorge C. Correia:** Writing – review & editing, Investigation. **Jonathan G. Van Vranken:** Writing – review & editing, Investigation. **Gabriel Ocana-Santero:** Writing – review & editing, Investigation. **Margareta Porsmyr-Palmertz:** Project administration, Investigation. **Sarah McCann Haworth:** Writing – review & editing, Investigation. **Vicente Martínez-Redondo:** Writing – review & editing, Investigation. **Zhengye Liu:** Writing – review & editing, Investigation. **Mattias Carlström:** Writing – review & editing, Supervision, Funding acquisition. **Matthias Mann:** Writing – review & editing, Supervision, Funding acquisition. **Johanna T. Lanner:** Writing – review & editing, Supervision, Funding acquisition. **Ana I. Teixeira:** Writing – review & editing, Supervision, Funding acquisition. **Lars Maegdefessel:** Writing – review & editing, Supervision, Funding acquisition. **Bruce M. Spiegelman:** Writing – review & editing, Supervision, Funding acquisition. **Jorge L. Ruas:** Writing – review & editing, Writing – original draft, Supervision, Project administration, Funding acquisition, Conceptualization.

## Declaration of competing interest

The authors declare that they have no known competing financial interests or personal relationships that could have appeared to influence the work reported in this paper.

## Data Availability

The cover letter includes important information for reviewers' access to deposited data.
