# Supplementary figures and images for: Mustn1 is a smooth muscle cell-secreted microprotein that modulates skeletal muscle extracellular matrix composition
Source: Mol Metab. 2024 Mar 6;82:101912. doi: 10.1016/j.molmet.2024.101912 (PMC10950823; doi:10.1016/j.molmet.2024.101912)

Supplementary Figure 1 (related to Figure 2)

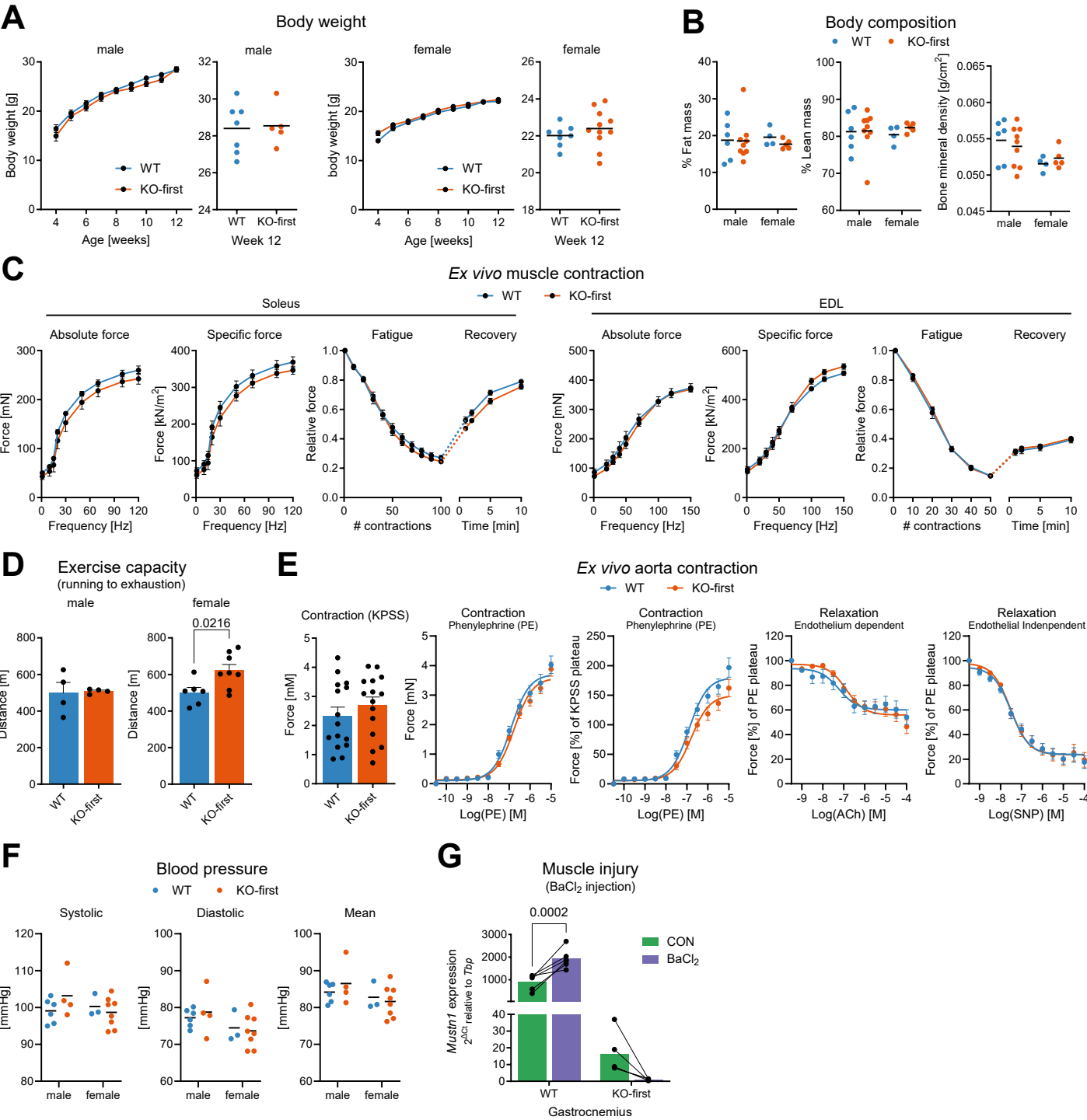

Supplement: Multimedia component 7 [file mmc7.pdf]

Supplementary Figure 2 (related to Figure 5D)

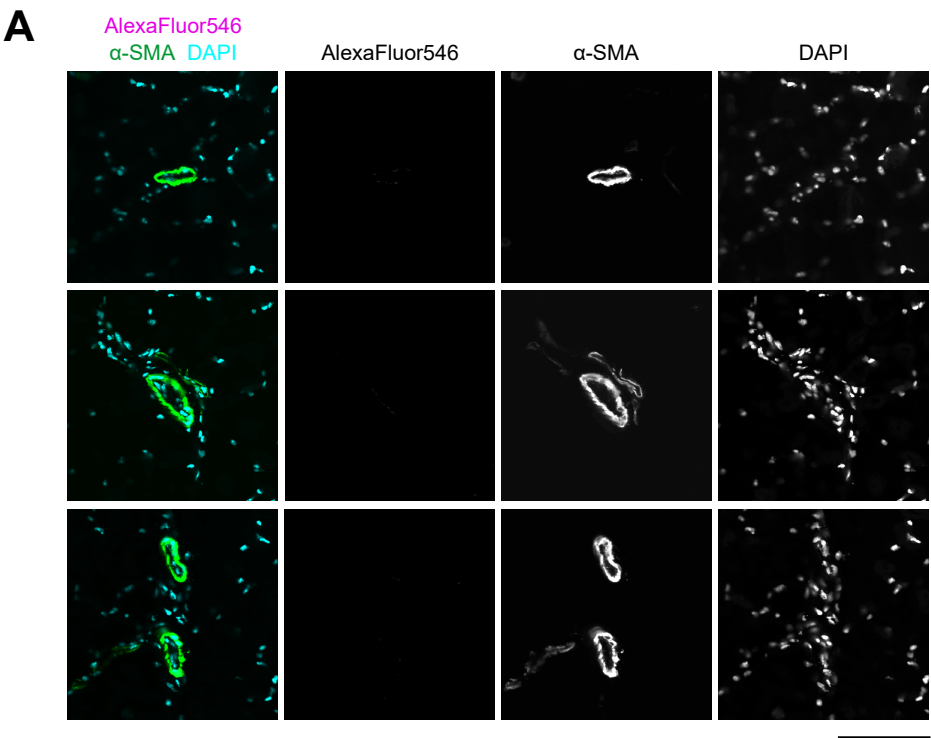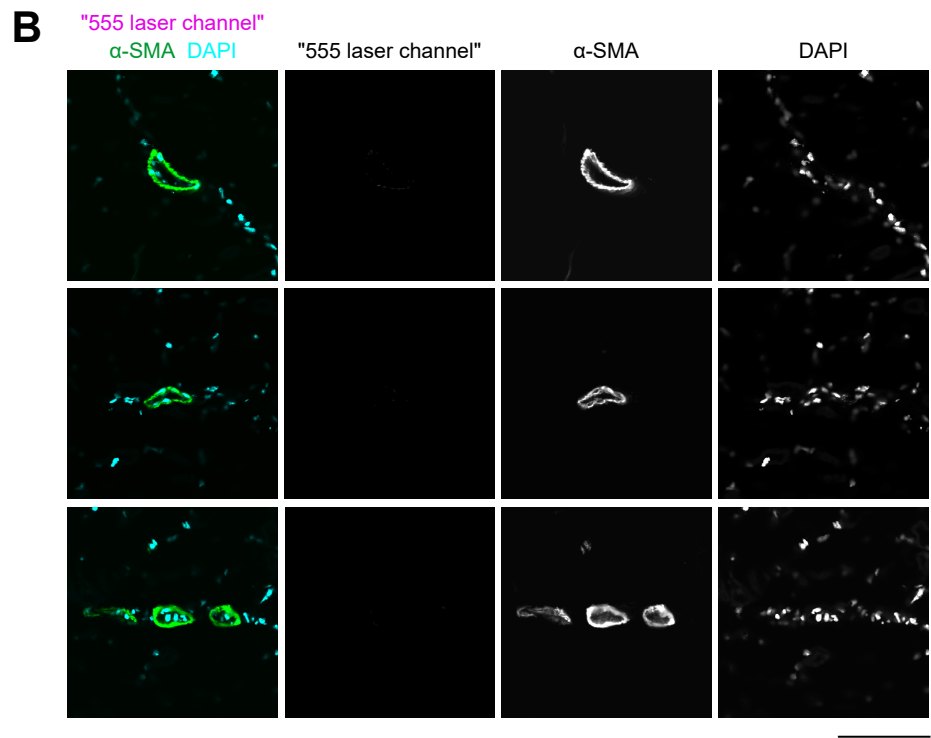

Supplement: Multimedia component 8 [file mmc8.pdf]

Supplementary Figure 3 (related to Figure 5E and F)

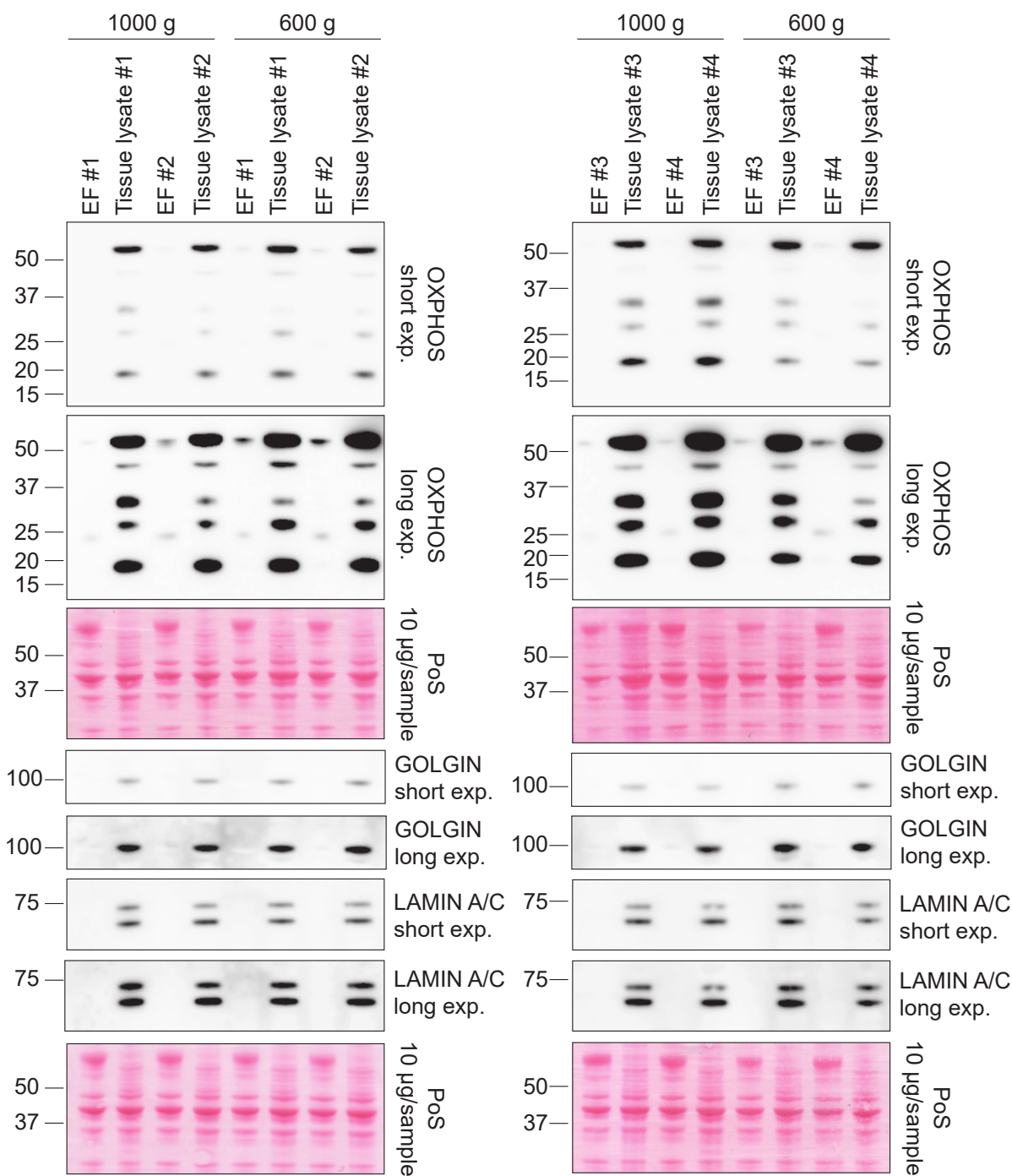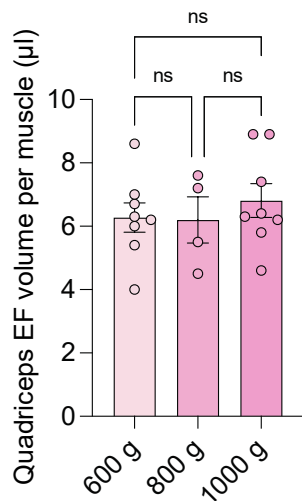

Supplement: Multimedia component 9 [file mmc9.pdf]
